# Current clinical applications of AAV-mediated gene therapy

**DOI:** 10.1016/j.ymthe.2025.04.045

**Published:** 2025-05-05

**Authors:** Barry J. Byrne, Kevin M. Flanigan, Susan E. Matesanz, Richard S. Finkel, Megan A. Waldrop, Eleonora S. D'Ambrosio, Nicholas E. Johnson, Barbara K. Smith, Carsten Bönnemann, Sean Carrig, Joseph W. Rossano, Barry Greenberg, Laura Lalaguna, Enrique Lara-Pezzi, Sub Subramony, Manuela Corti, Claudia Mercado-Rodriguez, Carmen Leon-Astudillo, Rebecca Ahrens-Nicklas, Diana Bharucha-Goebel, Guangping Gao, Dominic J. Gessler, Wuh-Liang Hwu, Yin-Hsiu Chien, Ni-Chung Lee, Sanford L. Boye, Shannon E. Boye, Lindsey A. George

**Affiliations:** 1 Powell Gene Therapy Center, University of Florida College of Medicine, Gainesville, FL, USA; 2 Center for Gene Therapy, Nationwide Children’s Hospital, Columbus, OH, USA; 3 Department of Pediatrics and Neurology, The Ohio State University, Columbus, OH, USA; 4 Clinical In Vivo Gene Therapy, Children’s Hospital of Philadelphia, Philadelphia, PA, USA; 5 Department of Neurology, University of Pennsylvania School of Medicine, Philadelphia, Philadelphia, PA, USA; 6 Center for Experimental Neurotherapeutics, St. Jude Children’s Research Hospital, Memphis, TN, USA; 7 Department of Neurology, University of Massachusetts Chan School of Medicine, Worcester, MA, USA; 8 Department of Neurology and Center for Inherited Muscle Research, Virginia Commonwealth University, Richmond, VA, USA; 9 Department of Physical Therapy, College of Public Health and Health Professions, University of Florida Gainesville, FL, USA; 10 Department of Pediatrics, University of Florida College of Medicine, Gainesville, FL, USA; 11 Neuromuscular and Neurogenetic Disorders of Childhood, NINDS/NIH, Bethesda, MD, USA; 12 Department of Pediatrics, University of Pennsylvania School of Medicine, Philadelphia, PA, USA; 13 Division of Cardiology, Children’s Hospital of Philadelphia, Philadelphia PA, USA; 14 Cardiology Department, University of California, California, San Diego, La Jolla, CA, USA; 15 Centro Nacional de Investigaciones Cardiovasculares Carlos III (CNIC), Madrid, Spain; 16 Centro de Investigación Biomédica en Red Cardiovascular (CIBERCV), Madrid, Spain; 17 Department of Neurology, University of Florida College of Medicinem, Gainesville, FL, USA; 18 Division of Neurology, Children’s National Hospital, Washington, DC, USA; 19 Department of Genetic and Cellular Medicine, Horae Gene Therapy Center, University of Massachusetts Chan School of Medicine, Worcester, MA, USA; 20 Department of Medical Genetics and Pediatrics, National Taiwan University Hospital, and College of Medicine, National Taiwan University, Taipei, Taiwan; 21 Center for Precision Medicine, China Medical University Hospital, Taichung, Taiwan; 22 Division of Cellular and Molecular Therapy, Department of Pediatrics, University of Florida College of Medicine, Gainesville, FL, USA

**Keywords:** *in vivo* gene therapy, adeno-associated viral vectors

## Abstract

Currently, there are an estimated 8,000 genetic disorders that cumulatively affect approximately 10% of the population. Even among the 5% of patients with genetic disease that have treatment options, these therapeutics rarely address the underlying cause of disease but rather focus on managing or modifying symptoms and typically require recurrent, lifelong therapy. A therapeutic approach to genetic disease that *in vivo* delivers a functional copy of the aberrant gene is an intuitive solution that has thus far taken 3 decades to reduce to clinical practice, predominantly using adeno-associated viral (AAV) vectors. Among available viral and non-viral gene delivery approaches, AAV vectors remain the most efficient means for *in vivo* delivery of DNA to the nucleus. AAV vectors now constitute a *bone fide* novel therapeutic drug class composed of seven US Food and Drug Administration-approved products with over 10-fold more in clinical development for an expanding number of disease indications and an identified list of problems to overcome for widespread clinical application. Here, we review current progress in clinical AAV gene therapy, including for neuromuscular disorders, hemophilia, primary cardiovascular disorders, or disorders with cardiovascular manifestations, lysosomal storage disorders, mucopolysaccharide disorders, primary central nervous systemic disorders, and ocular disorders.

## Graphical abstract

**Figure:**
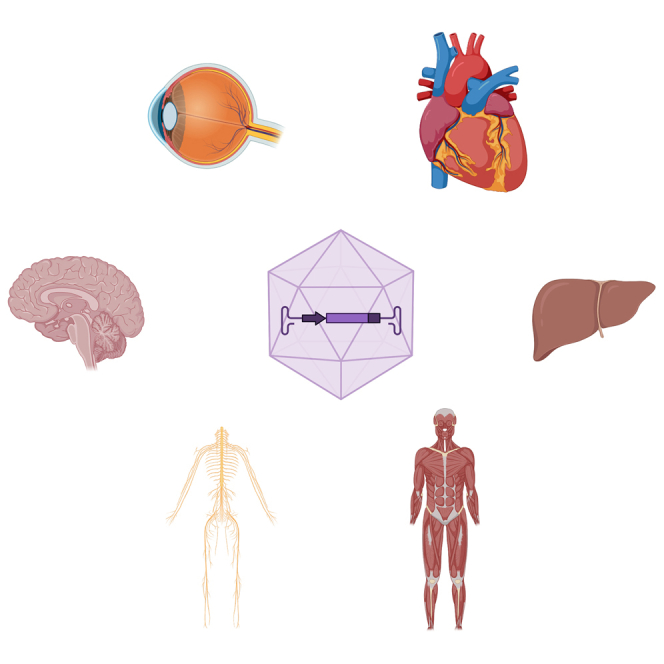

## Teaser

This review captures the most comprehensive and up-to-date information in the rapidly advancing field of AAV gene therapy, highlighting therapeutic strategies, clinical outcomes, safety concerns, and emerging innovations in vector design and gene editing across diverse inherited disorders, offering insights into future advancements and potential clinical applications.

## Introduction

There are >10,000 described rare disorders worldwide, among which 80% are genetic in origin, and only an estimated 5% of them have available therapies. Cumulatively, these 8,000 genetic disorders affect roughly 10% of the population, making rare genetic diseases a public health concern.^1^ Among patients with single gene or monogenic disorders, approximately two-thirds are children, accounting for 45% of pediatric healthcare claims.^2^ This underscores the potential impact of gene-based therapies for children and that pediatric patients incur the brunt of genetic illness. Even among the 5% of patients with genetic disorders that have available treatment, outside of the few recently licensed gene therapies, nearly none of these therapeutics address the underlying cause of disease, but instead aim to manage or modify symptoms and typically require recurrent, lifelong drug administration. A therapeutic approach to genetic illness that provides a functional copy of the aberrant gene or corrects the genetic defect is an intuitive solution that has so far taken 3 decades to implement as a clinical success in select disorders, many of which are highlighted herein.

Recombinant adeno-associated viral (AAV) vectors are a class of viral vectors used in gene therapy to *in vivo* deliver genetic material to targeted transduced cells. Recombinant AAV vectors are engineered from wild-type AAV (wtAAV), which is a replication incompetent, non-pathogenic virus that is a member of the Parvoviridae family. Recombinant AAV vectors lack wtAAV protein-coding sequences and instead incorporate an expression cassette consisting of the transgene, regulatory elements, and inverted terminal repeats (ITRs), the latter necessary for viral vector production and formation of stable episomal DNA. AAV vectors are predominantly non-integrating, forming extrachromosomal episomes in the nucleus with the potential for long-term gene expression.^3^ They are a favorable method of gene delivery due to their safety profile, and their ability to impart sustained episomal gene expression and to transduce a variety of cell types. Further, among available viral and non-viral gene delivery approaches, AAV vectors remain the most efficient means for *in vivo* delivery of DNA to the nucleus of a variety of cell types. This, combined with an established regulatory framework, manufacturing infrastructure, and proven clinical efficacy and safety, supports that AAV vectors will remain the leading choice for donor DNA delivery for *in vivo* gene addition and gene editing in the near and intermediate future, and possibly beyond. That said, AAV vectors are limited by the 4.7-kb cargo packaging capacity, limiting the size of donor template DNA, and in the case of gene addition, the requirement to target post mitotic, senescent cells. Further, although AAV has thus far demonstrated an excellent safety profile, observations at high doses, typically >1 × 10^14^ vg/kg, have revealed their potential to impart substantial toxicity from immunological responses to the vector.^4^^,^^5^^,^^6^^,^^7^ Last, low-frequency random integration events pose the theoretical risk of genotoxicity,^8^^,^^9^ but this has thus far not been clinically observed. Nonetheless, clinical success in AAV gene therapy has solidified AAV as a *bona fide* therapeutic drug class now composed of seven US Food and Drug Administration (FDA)-approved vectors (Table 1), and over 10-fold more in early and late-stage clinical development for an expanding number of disease indications and an identified list of problems to overcome for widespread clinical application. Here, we review the current status of AAV vectors for clinical *in vivo* gene therapy for genetic disorders.

**Table 1:** FDA-approved AAV vectors

Vector	Indication	Manufacturer	Administration route	Dose (×10^12^ vg/kg)	Capsid	Approval year
Voretigene neparvovec (Luxturna)	retinal dystrophy (mutation in RPE65)	Spark Therapeutics	subretinal injection	0.15 vg/eye	AAV2	2017
Onasemnogene abeparvovec (Zolgensma)	SMA	Novartis	intravenous	110	AAV9	2019
Etranacogene dezaparvovec (Hemgenix)	hemophilia B	CSL Behring	intravenous	20	AAV5	2022
Valoctocogene roxaparvovec (Roctavian)	hemophilia A	Biomarin	intravenous	60	AAV5	2023
Delandistrogene moxeparvovec (Elevidys)	DMD	Sarepta Therapeutics	intravenous	133	rh74	2023 (accelerated ages 4-5y) 2024
Fidanacogene elaparvovec (Beqvez)	hemophilia B	Pfizer	intravenous	0.5	rh74 variant	2024^a^
Eladocagene exuparvovec (Kebildi/Upstaza)	AADC	PTC Therapeutics	bilateral intraputaminal infusion	0.18	AAV2	2024

In 2024 delandistrogene moxeparvovec was granted full approval for ambulatory patients and accelerated approval for non-ambulatory patients. The remaining vectors have full FDA approval. The doses of voretigene neparvovec and eladocagene exuparvovec are denoted in vector genomes (vg) per eye or total dose, respectively. The remaining vector doses represent vg/kg. SMA, spinal muscular atrophy; DMD, Duchenne muscular dystrophy; AADC, Aromatic l-amino acid decarboxylase.

a Pfizer withdrew BEQVEZ from the market.

## Intravenous AAV gene therapy

The first clinical use of an AAV vector infused into circulation was reported in the early 2000s^10^ in a trial conducted for *F9* gene transfer for hemophilia B (HB). While the trial did not demonstrate sustained efficacy, the work provided the first safety data^10^ for intravenous AAV vector infusion, established a regulatory framework for clinical translation of AAV vectors, outlined efficacy limitations of preexisting AAV neutralizing antibodies^10^^,^^11^ as well as their persistence after dosing,^12^ demonstrated AAV is shed in semen for a duration that is vector dose dependent,^10^^,^^13^^,^^14^ and unmasked unexpected immunological responses to AAV that have been expanded in subsequent studies^5^^,^^6^^,^^15^^,^^16^^,^^17^^,^^18^^,^^19^^,^^20^^,^^21^ and continue to pose safety and efficacy limitations to AAV gene therapy.^22^ Currently, most AAV vectors are delivered by intravenous infusion, including five of the seven FDA-approved AAV vectors.

### Congenital neuromuscular disorders

Neuromuscular disorders affect components of the motor unit that encompass the peripheral nervous system, neuromuscular junction, and skeletal muscle. These disorders have no curative therapies and, in many cases, no meaningful therapeutic intervention. Currently, there are two FDA-approved products for congenital neuromuscular disorders: onasemnogene abeparvovec (Zolgensma, full approval in 2019) for type 1 spinal muscular atrophy and delandistrogene moxeparvove (Elevidys, initial accelerated approval in 2023 and full approval 2024) for Duchenne muscular dystrophy (DMD). Efforts translating AAV for congenital neuromuscular disorders have shown remarkable progress over the past decade and outlined a series of challenges to overcome for next generation efforts to build on current successes. A primary challenge for this class of disorders remains effective delivery to the target tissue(s), which has thus far necessitated using among the highest AAV vector doses employed in clinical AAV efforts, and unmasked likely dose-limiting toxicities of AAV that have, in rare circumstances, resulted in mortality.^23^ Additionally, in some cases, the natural history of the disorder may be unknown or altered by preliminary successes in gene therapy, underscoring the complexity of defining clinical trial endpoints and the importance of continued understanding of underlying and evolving disease pathology. Herein, we review the status of clinical AAV gene-therapy approaches for congenital neuromuscular disorders.

#### Duchenne muscular dystrophy

DMD is the most common genetic neuromuscular disorder in childhood, caused by mutations in the X-linked *DMD* gene, which encodes the 427-kD protein dystrophin. Dystrophin is a critical linkage protein of the contractile actin filaments to the sarcolemma of skeletal and smooth muscle cells, and other isoforms are expressed in the brain.^24^ Patients present in childhood with early motor delays, motor plateau, loss of motor skills, typically losing the ability to ambulate at age 10–13 years. As the disease advances, the lack of functional dystrophin leads to a cascade of inflammation, muscle fibrosis, impaired muscle regeneration, and fatty replacement that correlates with a progressive decline in motor function. Development of cardiomyopathy and progressive respiratory failure leads to death in the late teens to mid-twenties.^25^ Incidence is 1 in 5,000 male births,^25^^,^^26^^,^^27^ with an estimated 15,000 individuals in the United States and 300,000 individuals globally living with DMD.^28^ Given the large number of affected individuals and the high burden of disease, DMD has been a strong target for therapeutic development.

In recent years, there has been an increase in therapeutic options for DMD. Antisense oligonucleotide (AON) exon-skipping therapies have been on the commercial market since 2016 using the accelerated approval pathway, but efficiency of delivery has yielded only limited increases in dystrophin production.^29^ These treatments are mutation-specific and available to only approximately 30% of DMD patients. Second-generation therapies, designed to increase protein production through more efficient muscle cell delivery, are currently in clinical trials and varying stages of preclinical development.^30^ In 2023, vamorolone, a dissociative steroid, was approved for use in DMD. Shortly thereafter in 2024, ginivostat, a histone deacetylase inhibitor, was also approved for use in those older than 6 years, regardless of genetic subtype.

**Figure 1:**
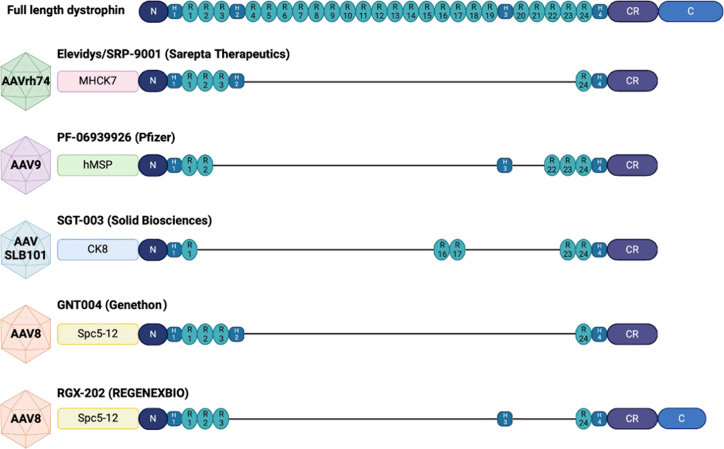
Current micro-dystrophin constructs and functional domains vs. full-length dystrophin

Given the large size of the dystrophin protein and the 4.7-kb optimum packing capacity of AAV vectors, clinical development has focused on micro-dystrophin constructs. The concept of micro-dystrophin as a therapeutic target was modeled after a Becker muscular dystrophy patient with a large in-frame dystrophin deletion who remained ambulatory into his 60s. To date, five different versions of micro-dystrophin have been studied in clinical trials sponsored by Sarepta Therapeutics, Pfizer, Solid Biosciences, RegenxBIO, and Genethon^31^ (Figure 1). All programs require increased doses of corticosteroids in the peri-dosing window to decrease immune-mediated side effects.

Sarepta’s delandistrogene moxeparvovec (Elevidys) became the first commercially approved micro-dystrophin in June of 2023 when it was granted accelerated approval for 4- and 5-year-olds. The product uses a micro-dystrophin cDNA and MHCK7 promoter in the AAVrh74 capsid. This approval yielded significant optimism for a class of therapies that may be broadly applicable. Initial accelerated approval in 4- and 5-year-olds was based on the results of study 102, a phase 2, double-blind, randomized control trial (RCT) of 41 males ages 4 to <8 and AAVrh74 titers <1:400. All participants were required to have an out-of-frame mutation between exons 18 and 58, and to be on a stable dose of corticosteroids for at least 12 weeks. The trial did not meet its primary efficacy endpoint, change in North Star Ambulatory Assessment (NSAA) score at 52 weeks, but did show benefit in the subgroup of 4- and 5-year-old patients.

In June 2024, delandistrogene moxeparvovec obtained full approval for all ambulatory patients and accelerated approval for non-ambulatory patients. This expanded approval was not without controversy; as documented in the FDA’s Biologics License Application (BLA) Integrated Clinical and Clinical Pharmacology Review Memorandum, the clinical and clinical pharmacology reviewers recommended against label expansion, a decision overridden by the CBER divisional director.^32^^,^^33^ The confirmatory randomized controlled trial, study 301 (NCT05096221, EMBARK)^34^ included 125 boys ages 4 to <8, with an NSAA score of >16 to <29, and time-to-rise (TTR) of <5 s. Subjects were matched based on both age and baseline NSAA score and followed for 52 weeks. Notably, the study did not meet its primary endpoint, change in NSAA. Boys who received delandistrogene moxeparvovec improved 2.6 points on the NSAA compared with those who received placebo (*p* = 0.24), with benefit on key secondary outcomes seen on 10-m walk-run and TTR, among other secondary outcome measures. Because the primary endpoint was not met, statistical significance from the secondary outcome measures cannot be drawn.^34^ Mean micro-dystrophin expression was 34.3%, and median was 19.1%^34^; neither individual subject expression data nor a range of values was reported, but the FDA’s analysis showed no correlation between dystrophin expression and function.^34^ Overall, the medication was well tolerated, with serious adverse events (SAEs) seen in seven patients (11.1%). There were five patients with liver-related SAEs, and one case of rhabdomyolysis based on creatine kinase (CK) levels and distinct from prior reports of immune-mediated myositis. There was also one case of myocarditis, presenting at 6 h after infusion with pyrexia, nausea, and vomiting. Troponin-I was elevated, peaking at 140 × upper limit of normal on day 2, and the myocarditis resolved by day 21 when echocardiogram and troponin-I were normal. Notably, there were no cases of thrombotic microangiopathy (TMA).^34^ A trial in non-ambulatory subjects, ENVISION (NCT05881408) is ongoing. This trial is actively enrolling participants, all of whom are required to have an ejection fraction of 40% and a forced vital capacity of 40%.

While the approval of delandistrogene moxeparvovec has yielded enthusiasm and marks a significant clinical milestone, questions remain about safety and efficacy. This is particularly the case in older DMD patients with greater disease-related comorbidities, particularly non-ambulatory patients. Combining clinical trials and commercially treated patients, approximately 800 patients have been treated with Elevidys as of January 2025. The first Elevidys-related death was announced in March 2025, in a 16-year-old non-ambulatory male secondary to acute liver failure presenting around 9 weeks after gene transfer.^35^ Additionally, the authors are aware of three deaths from cardiac-related events that have occurred between 2 and 5 years post vector. The relationship of these events to the product has not yet been fully evalauted. Treatment decisions require detailed discussions of an individual’s potential benefit and risk and the failure of the therapy to meet its defined endpoint in the confirmatory trial leaves room for some clinical equipoise in considering enrollment in ongoing trials of alternate micro-dystrophin regimens. Durability of effect also remains an open question. Longer-term data are only available from the first four patients, treated at a mean age of 5.1 years in an open-label study, who notably showed much higher levels of micro-dystrophin expression (a mean of 74.3%) than those in the EMBARK randomized trial.^36^ Evidence for durability was seen at 4 years post gene transfer, at which point these subjects showed a mean increase in NSAA of 7 points.^37^ Given the small sample size, additional data are needed on duration of therapeutic benefit, although there are patient reports suggesting limited durability beyond 2 years, a long-term follow-up study of early treated patients is ongoing (NCT05967351).

Emergence of unexpected side effects remains a challenge in several AAV programs. After five cases of myositis were seen across three clinical programs (Sarepta, Pfizer, and Genethon), cooperation across development programs led to a tightening of clinical trial entrance criteria based on mutation location. Cases occurred 3 to 6 weeks after dosing, consistent with an anti-transgene response. Three of the five also had co-occurring myocarditis. All patients had deletions encompassing exons 8–11, corresponding to hinge 1 and the beginning of the spectrin repeat domain, an area contained in all the transgene constructs (Figure 1). Thus, the hypothesis is that a cytotoxic T cell response occurred as a reaction to non-self-epitopes in the micro-dystrophin protein.^38^ Subsequent trials have mostly excluded those with early deletions, with the range of restrictions varying across clinical development programs.

Pfizer’s program, utilizing an AAV9 serotype and a muscle-specific promoter, was discontinued after failure of its product, fordadistrogene movaparvovec, to meet clinical endpoints in the phase 3 RCT CIFFREO trial (NCT04281485) with prior reported major safety concerns, including mortality events. CIFFREO enrolled 99 boys ages 4 to <8 who received a dose of 2 × 10^14^ vg/kg. The company announced in June of 2024 that it did not meet its primary endpoint, change in NSAA, or key secondary endpoints, including TTR and 10-m walk/run test.^39^ The failure to meet clinical endpoints occurred despite production of micro-dystrophin (mean of 52.1%).^40^ In the cohort, four cases of myocarditis and three cases of TMA were reported.^40^ This news followed the announcement in May 2024 of a death due to recurrent rhabdomyolysis and hyperkalemic cardiac arrest in a participant in the phase 2 DAYLIGHT trial, an open-label study of young boys ages 2 to 4^41^ (NCT05429372). Additional details about the death, which occurred more than a year after dosing, have not been released. This was the second death in the program, with the first occurring in December 2021 in a non-ambulatory 16-year-old participant secondary to myocardial inflammation and severe left ventricular dysfunction 6 days after treatment in the phase 1b trial (NCT03362502).^42^ The subject experienced rising troponin levels and cardiogenic shock. Autopsy was not done, and the death was hypothesized to be due to an innate immune response.^42^ The Pfizer program also reported cases of TMA/aHUS (atypical hemolytic uremic syndrome), occurring within 10 days of vector administration.^42^ Whether the greater toxicities observed with fordadistrogene movaparvovec relative to delandistrogene moxeparvovec are due to differences in vector capsid, enrolled patient cohorts, dystrophin transgene cassette sequence, some combination thereof, or other causes is not known.

Solid Biosciences also has an active clinical development program. A first-generation therapy, SGT-001 (NCT03368742), enrolled nine patients (six of nine at a high dose of 2 × 10^14^ vg/kg) in a phase 1/2 open-label clinical trial, IGNITE DMD, using AAV9 and the CK8 muscle-specific promoter. The expression cassette contains the neuronal nitric oxide synthase (nNOS)-binding domain, encoded for by SRs R16-R17. SGT-001 was subject to two separate clinical holds, in 2018 and 2019, secondary to complement-mediated events.^43^ While the hold was lifted in 2020 after manufacturing issues, including reducing empty capsids, were addressed, additional patients were not treated through IGNITE DMD. Three-year follow-up data of the initial nine patients demonstrated maintenance of motor function and no new safety signals.^44^ Solid’s second-generation gene therapy program, SGT-003, uses a novel adeno-associated virus vector (AAV-SLB101) with a goal of enhanced muscle delivery and decreased liver tropism at a lower dose of 1 × 10^14^ vg/kg. The phase 1/2 INSPIRE DMD trial is currently enrolling ambulatory males with DMD on stable doses of corticosteroids, in two cohorts, ages 4 to <7 and 7 to <12 meeting certain functional requirements (NCT06138639). Animal data using SGT-003 presented at the 2024 American Society of Gene & Cell Therapy annual meeting showed micro-dystrophin protein localization as well as nNOS activity, with localization to the sarcolemma, as well as evidence of functional improvement and reduction in certain biomarkers.^45^

REGENXBIO’s product RGX-202 utilizes an AAV8 vector, an Spc5-12 promoter, and includes functional elements of the C-terminal (CT) domain. A phase 1/2 trial is ongoing, AFFINITY DMD (NCT05693142). Enrolled participants had mutations in exons 18 or above. To date, three boys ages 4 to <11 received a lower dose of 1 × 10^14^ vg/kg and four have received a higher dose of 2 × 10^14^ vg/kg. Muscle biopsy data at 10 weeks showed micro-dystrophin expression ranging from 11% to 83%, with reduction in CK of 43%–90%.^46^ In the seven patients treated thus far, there have been no serious treatment-related adverse events (AEs) as of July 2024. AFFINITY DMD will enroll up to 12 patients at the second dose, including up to five patients ages 1 to 3.

Genethon’s candidate, GNT0004, also utilizes an AAV8 vector with an Spc5-12 promoter. A phase 1/2/3 trial is ongoing in France and the United Kingdom (Eudra CT, 2020-002093-27), enrolling boys with DMD ages 6 to 10. The initial five patients were treated in two dose cohorts, *n* = 2 received a dose of 1 × 10^13^ vg/kg and *n* = 3 received 3 × 10^13^ vg/kg. The patients in the higher dose cohort showed mean micro-dystrophin expression of 54% at week 8, and a decrease in CK of 50%–87% at week 16.^47^ Direct comparison of the protein expression results among these micro-dystrophin trials is difficult, as variation may be present in the vector dosing (e.g., due to differences in titering methods) or in the dystrophin quantification protocol for each.

In addition to micro-dystrophin, other approaches of gene correction have been attempted in DMD. Most notably, an *n* = 1 trial utilized a CRISPR-transactivator approach. A 27-year-old with DMD received 1 × 10^14^ vg/kg of rAAV9 containing d*Sa*Cas9 fused to VP64, which was designed to upregulate a non-muscle, cortical, isoform of dystrophin (Dp427c). He was pretreated with a single dose of rituximab (400 mg) −13 days before treatment and received methylprednisolone 10 mg/kg on day −1 and day 0. Sirolimus 1 mg/kg was also initiated at day −1, and he was then started on prednisone 2 mg/kg on day +1 after infusion. Symptoms developed in the days following infusion, including worsening cardiac function with pericardial effusion on day 5 and respiratory distress on day 6. Additional immune therapies were added, including higher doses of steroids, eculizumab, tocilizumab, and anakinra, but symptoms progressed, and the patient suffered a cardiac arrest and died 8 days after treatment. On autopsy, there was minimal transgene expression and no evidence of AAV9 antibodies, and the death is hypothesized to be due to an innate immune response leading to a cytokine-mediated capillary leak syndrome,^48^ raising concerns around the ability of patients with advanced cardiac disease to tolerate AAV-related toxicities observed at vector doses currently used for DMD. Although in this case the time course favored an innate immune response to the capsid as the primary pathogenic event, an innate response to expression of the potentially immunogenic viral-derived VP64 cannot be excluded, and whether long-term expression of this transcriptional activator may lead to acquired immune responses remains to be seen.

While there is optimism around these approaches, challenges and questions remain about long-term efficacy and safety data of newly approved therapies. Additionally, there is need for more data on combination therapies and the potential of improved tissue-specific capsid tropism, and restoration of full-length, durable dystrophin remains to be the main goal. Toward this goal, the recent work from the Chamberlain group^49^ and Han group^50^ on expressing large dystrophins using protein *trans*-splicing mechanism (via split inteins) is quite promising. However, even though expression of larger midi-dystrophins or even full-length dystrophin may have better therapeutic benefit than micro-dystrophin expression, it may not be without risk. Full-length dystrophin will contain epitopes to which the majority of patients will not have previously been exposed, which may result in neoantigen-directed immune responses. In addition, the split intein approach results in release of the ligated intein peptide, which may be immunogenic.^49^ While current approaches have primarily utilized a gene replacement strategy, gene editing approaches, currently being studied, may represent a longer-term, more durable solution toward restoration of full-length dystrophin.^51^

#### Spinal muscular atrophy

Classic proximal spinal muscular atrophy (SMA) is an autosomal recessive disease affecting primarily motor neurons and resulting in progressive weakness with related pulmonary, bulbar, and musculoskeletal complications.^52^ The most severe form, type 1, presents in early infancy. These infants never achieve sitting, and survival is typically less than 2 years. The causative gene for SMA, *SMN1*, was identified in 1995, with biallelic deletions or pathogenic variants causing a deficiency of the survival motor neuron (SMN) protein, necessary for motor neuron development and survival.^53^ A paralogous “backup” gene, *SMN2*, rescues an otherwise lethal condition by supplying a small amount of SMN, although insufficient for normal motor neuron growth, development, and maintenance.^54^ Though not the sole predictor of phenotype, increasing copies of *SMN2* often correlate with a milder phenotype.^55^

Following discovery of the *SMN1* gene, mouse models were created. One notable example is the triple transgenic “delta-7” mouse by the Burghes lab, which has proven informative in drug development.^56^ Additionally, two gene-targeted treatment strategies emerged: replacement of the non-functional *SMN1* gene with a normal copy via intravenous delivery by an AAV-9 vector (initially “AVXS-101,” now onasemnogene abeparvovec, OA), and splicing-driven modulation of *SMN2* (nusinersen [periodic intrathecal delivery] and risdiplam [daily oral administration]), which increases production of full-length SMN.^57^^,^^58^^,^^59^ All three drugs have received regulatory approval in many countries after clinical trials demonstrated an overall strong safety profile and highly favorable efficacy, with improved survival and motor function, especially in patients treated early after symptom onset or even pre-symptomatically (usually when identified by newborn screening).^60^^,^^61^^,^^62^ This section focuses on Zolgensma (Onasemogene, OA).

The concept and development of a gene therapy vector for SMA arose in the Kaspar lab.^63^ This AAV9 vector includes a self-complementary construct with a chicken β-actin (CBA) promoter, cytomegalovirus (CMV) enhancer, and the full *SMN1* cDNA. Initial treatment on patient cells *in vitro* demonstrated an increase in SMN protein. Intravenous administration to the delta-7 mouse showed improved survival and motor function, and studies with cerebrospinal fluid (CSF) delivery enabled dose-escalation testing of safety and efficacy.^63^^,^^64^ Clinical development was then pursued by AveXis using the clinical grade vector manufactured at the Nationwide Children’s Hospital gene therapy facility. This vector was used in the phase 1/2A study led by Dr. Jerry Mendell.^57^ This vector crosses the blood-brain barrier and can therefore be administered intravenously as a single dose. This single-arm study in infants under 6 months of age, with a historical control cohort, was notable for the three low-dose (6.7 × 10^13^ vector genomes/kg) patients having stabilization but no clear improvement, while the 12 higher dose (2.0 × 10^14^ vg/kg) patients showed a remarkable response and strong safety profile. It was in that study, after administration of OA to the first patient, that significant transaminitis was identified, prompting subsequent patients to be pretreated with prednisone at 1 mg/kg/d, and maintained at that dose for the next month and then tapered. Post licensure, the label includes a boxed safety warning for liver toxicity. These patients continue to do well with sustained durability of effect at least 5 years after dosing (and 9 years in some cases).^65^^,^^66^

Widespread target engagement in motor neurons has been demonstrated subsequently in postmortem studies from individuals in other trial cohorts.^67^ The manufacturing process was changed to a more commercial grade product and resulted in a different dose (1.1 × 10^14^ vg/kg) administered in subsequent studies and is now the recommended commercial dose. The phase 1/2A study, with support from phase 3 studies then in progress,^68^^,^^69^ prompted a rapid review by the FDA and approval of a single dose administered intravenously in patients with genetically confirmed SMA up to 2 years of age in the United States. The EMA approval for Europe is different, allowing treatment up to 21 kg weight in patients of any age with type 1 SMA or up to three copies of *SMN2*. With the advent of newborn screening (now universal in the United States and evolving in several other countries), patients were identified in the pre-symptomatic or early symptomatic state.^70^^,^^71^ A clinical trial in these infants under 6 weeks of age demonstrated an even more robust response, with most of the patients with three copies of *SMN2* showing typical growth and development, and those with two copies being generally healthy and making remarkable gains in motor function, but often lagging behind typically developing children.^20^^,^^61^

OA is now available in over 60 countries, and more than 4,000 patients have been treated with the commercial product. Some of these patients have been enrolled in real-world evidence registries and reported in case series. Data are now available on a wider population of patients including those who are older, heavier, and/or have one and four copies of *SMN2*.^66^^,^^72^^,^^73^^,^^74^ Nearly all patients treated with OA sustain a transient adverse event (fever, vomiting), usually within days to weeks of administration. Transaminitis is seen in up to 90% of patients and in rare cases has led to frank liver failure and death.^66^^,^^75^^,^^76^^,^^77^ Liver biopsy data of two patients who developed liver failure 4–8 weeks post OA demonstrated CD8+ T cell infiltration on immunohistochemistry,^5^ suggesting the toxicity followed a pattern consistent with an immune response to the AAV capsid previously reported in hemophilia patients post AAV.^22^ Thrombocytopenia is commonly seen in the first week after administration of OA, more rarely thrombotic microangiopathy (TMA, atypical hemolytic uremic syndrome) has occurred, including events with associated mortality.^19^^,^^78^^,^^79^ Uncommonly, patients have suffered from severe and even fatal super-infection from other viruses when immunosuppressed with prednisone.^67^ Elevated troponin-I levels are often noted in neonates with SMA pretreatment and do not appear to predict cardiac dysfunction after administration of OA, although regular monitoring of this biomarker is still recommended.^80^ Other rare serious adverse events have been reported, e.g., necrotizing enterocolitis.^81^ Approximately 10%–50% sustain a serious adverse event that may require hospitalization.^76^ These are typically predictable and manageable. In one European study of individual case safety reports there were 39 fatalities among 661 cases (5.9%) attributed to cardiac arrest, respiratory failure or hepatic failure, with 90% having received OA monotherapy.^82^ This highlights the importance of being vigilant with standard-of-care support and close monitoring of the patient after administration of OA. The overall safety profile for OA remains strong, with over 98% of patients tolerating the administration of OA and immune suppression with prednisone without sustained serious or fatal side effects.^76^^,^^82^ A thorough analysis of data from 325 individuals with SMA who received OA through clinical trials, managed access programs, or commercial use revealed a mean prednisone course duration of 83 days (range 33–229 days).^83^

These observations have led to evolving recommendations for identification of risk factors when patients should not be treated, e.g., elevated anti-AAV9 antibodies or acute infection, as well as guidelines for monitoring and treating patients after administration of OA. The Novartis Prescribing Information provides current recommendations for monitoring.^84^ Many of these patients are treated in centers of excellence that have the expertise to monitor and react promptly to adverse events that might arise.^85^

To mitigate viral load and dose in a broader range of individuals, intrathecal OA treatment in sitting/non-ambulant patients 6–60 months of age was explored with three fixed doses (NCT03381729).^86^ This study was terminated after studies in mice and non-human primates (with a higher dose vector having a different promoter) identified a risk of dorsal root ganglia (DRG) toxicity.^87^^,^^88^^,^^89^ After a thorough review, no DRG toxicity was seen in the treated patients, and a new study (NCT05386680) was initiated to better monitor for DRG toxicity. Two patients in that study developed acute sensory symptoms suggestive of DRG toxicity.^90^ Otherwise, to date, no DRG-related toxicity has been identified in single cases or from safety registry reports.^82^

Combination treatment for SMA is now under active investigation, often empirically conducted by clinicians but also in a few clinical trials (NCT05522361, NCT05156320, NCT05861986, NCT05115110). Combination typically occurs in two scenarios: (1) initiate treatment with nusinersen or risdiplam (such as when there is an elevated antibody level) followed by eventual administration of OA, or (2) treatment with nusinersen or risdiplam following administration of OA when the response to OA is felt to be suboptimal. The safety and efficacy of this combined treatment has not yet been well defined.

OA is a costly drug with a list price of $2.1 million in the United States. Cost-effectiveness is hard to define in the context of substantial clinical benefit.^91^^,^^92^ It is not surprising that health authorities and payers in different countries have adopted different coverage policies, sometimes restricted to patients with two or three copies of *SMN2*, or only type-1 patients. A recent European consensus statement has made recommendation on therapy for SMA with OA.^93^ These policies will continue to be revised as new data emerge, especially that of treating pre-symptomatic patients identified by newborn screening. Payers are also considering alternative reimbursement policies like value-based payment plans to help with costs.^94^^,^^95^ Due to cost, global access to OA remains limited.^96^ Like all expensive medicines, individuals residing in wealthier countries are more likely to have access, whereas individuals living in lower income countries may only have access to these drugs through a very limited lottery system. Ethical considerations are also under active discussion.^97^

There are other genetic causes of motor neuron diseases that begin in infancy or childhood, under the umbrella of “SMA”. One example that has a gene replacement therapy under investigation is Spinal muscular atrophy with respiratory distress 1 (SMARD1).^98^ This disease is caused by autosomal recessive pathogenic variants in the *IGHMBP2* gene. Reduction in the immunoglobulin mu DNA binding protein 2 results in neuronal degeneration that is both length dependent and non-length dependent. Children with SMARD1 typically present in respiratory failure within the first few months of life, often after an illness or sedation event. Without significant feeding and respiratory support, the majority will not survive over 1 year of age. A pilot clinical trial utilizing an AAV9 vector (*n* = 7) is currently under way (NCT05152823).

#### Limb-girdle muscular dystrophy

Limb-girdle muscular dystrophies (LGMDs) are inherited muscle disorders characterized by progressive weakness. While these disorders were first identified in the early 19th century, they were fully described in 1954 by John Walton and F.J. Nattras.^99^ Advances in molecular biology in the 1990s led to the identification of specific genes associated with each LGMD subtype. The LGMD classification has been recently revised and in the new nomenclature dominant forms are designated as LGMDD and recessive as LGMDR.^100^

The LGMDs have an estimated worldwide prevalence ranging from 0.8 to 6.9 cases per 100,000. While there is regional variation in prevalence, LGMDR12, LGMDR1, and LGMDR2 are the most prevalent. LGMDs are characterized by proximal, slowly progressive muscle weakness, primarily affecting the shoulder and pelvic girdles. Patients can develop respiratory failure later in the disease course. Cardiac involvement is generally not a hallmark of LGMDs, with the exception of some sarcoglycanopathies (LGMDR4 and R6), the FKRP-related and the telethonin-related LGMDs (LGMDR9 and R7, respectively).^101^^,^^102^ Treatment for LGMDs is mostly supportive, as there are no known disease-modifying therapies. However, research has opened new possibilities, especially in the field of gene therapy. Gene replacement therapies have been successful in preclinical and clinical studies in many recessive LGMD subtypes, while the dominant forms may benefit from gene silencing approaches.^103^

Antisense oligonucleotide (ASO) therapy uses short, synthetic strands of nucleotides to target and alter expression of messenger RNA through various mechanisms (translation inhibition, splicing alteration, RNA degradation). In LGMDs, exon skipping using ASOs was tested effectively in fibroblasts of patients with mutations in the dysferlin gene (LGMDR2/2B). This group focused on exon 32 skipping, having shown that patients with an in-frame deletion of exon 32 exhibit a milder phenotype.^104^ Demonbreun et al. applied the multi-exon-skipping technique in a CRISPR-engineered mouse model of LGMDR5/2C. The group demonstrated that intramuscular administration of their multiple exon-directed ASOs resulted in the production of a functional protein and restored the expression of γ-sarcoglycan.^105^ In LGMDD1, investigators have used an allele-specific knockdown approach to reduce the expression of the DNAJB6 allele with toxic gain of function.^106^ Other dominantly inherited LGMDs may benefit from this approach.

The challenge with AAV gene therapy for neuromuscular disorders is to achieve high muscle tropism, while minimizing hepatoxicity and immune responses triggered by viral vectors.^107^ A gene therapy approach for LGMDs is highly viable because at least 15 LGMD subtypes have a gene small enough to be entirely packaged into an AAV vector. This enables stable expression of the full-length gene. Such approach mirrors the successful experience seen in SMA gene therapy, as opposed to the need for miniaturized gene versions, which is a current requirement for DMD treatment.^108^^,^^109^ Additionally, most of the mutations associated with LGMDs are missense mutations, which typically result in proteins that are present but altered in function. While the overall expression of the protein is potentially preserved, it can also pose significant issues and contribute to toxicity or trigger an adverse immune response *in vivo*. Over the years, various research groups have addressed different LGMD subtypes, as detailed below.

##### LGMDR1/2A (calpain)

In 2006, Bartoli et al. used an AAV1/2 vector with a muscle-specific promoter to deliver CAPN3 to the soleus muscle of a mouse model and observed increased muscle strength and size.^110^ However, when the same group attempted an intravenous delivery, they faced cardiac toxicity and eventually death of the animal. To address these complications, subsequent studies utilized a muscle-specific promoter that included the CAPN3 promoter and a target sequence of the cardiac-specific microRNA-208a. This strategy resulted in the stable expression of the transgene in the skeletal tissue, while suppressing expression in the heart.^111^^,^^112^

##### LGMDR2/2B (dysferlin)

Unlike calpain, dysferlin transcript’s size (6.9 kb) exceeds the AAV vector packaging capacity. To overcome this limitation, dual vectors have been designed and tested first intramuscularly and then intravenously in mice.^113^^,^^114^ Further studies of the dual vector via intramuscular or regional vascular delivery in both mice and non-human primates demonstrated full-length dysferlin expression and no safety concerns.^115^ This paved the way for an open-label, phase 1, single dose, intravenous gene transfer study of rAAVrh74. MHCK7.DYSF.DV vector in two ambulatory patients (NCT05906251).

##### LGMDR3/2D, LGMDR4/2E, LGMDR5/2C (α-,β-,γ-sarcoglycans, respectively)

These subtypes of LGMD represent potential gene therapy targets, particularly because the four transmembrane glycoproteins involved are relatively small. Preclinical studies in mice demonstrated that intramuscular delivery of a full-length cDNA was feasible and did not generate toxicity.^116^^,^^117^^,^^118^ The first-in-human study was an intramuscular injection of rAAV1.tMCK.hSGCA into the extensor digitorum brevis of six LGMDR3/2D patients. The trial demonstrated stable expression of the transgene and successful restoration of the sarcoglycan complex in all but one patient, who had preexisting antibodies against the AAV.^119^ Intravenous delivery was successful in mouse models of the sarcoglycanopathies, and guided subsequent in-human studies.^120^^,^^121^^,^^122^^,^^123^ A phase 1, multicenter, open-label, single dose, intravenous gene transfer study is ongoing to evaluate the safety, tolerability, and efficacy of rAAVrh74.MHCK7.hSGCB both in adults (NCT05876780) and children (NCT03652259). A novel approach to treating LGMDR3/2D, involves using cystic fibrosis transmembrane regulator correctors to restore defective α-sarcoglycan protein. This method has led to a clear improvement in muscle force in the treated mouse model.^124^

##### LGMDR9/2I (fukutin)

The loss of FKRP leads to defects in glycosylation of the α-dystroglycan. Preclinical trials addressing two different mutations in the FKRP gene showed positive results, both in terms of restoration of α-dystroglycan (αDG) glycosylation and improvement of dystrophic symptoms.^125^^,^^126^ Interestingly in another mouse model, higher injections of the vector (rAAV2/9 in this case) induced a decrease of αDG glycosylation and a dose-dependent toxicity, suggesting the need for a tightly regulating FKRP gene expression in muscles.^127^ Two gene replacement clinical trials on ambulatory patients are currently recruiting (NCT05230459 and NCT05224505). Promising data from Cataldi’s group highlighted the potential of combining AAV gene therapy for LGMDR9/2I with ribitol. The hypothesis is that ribitol, a sugar alcohol, acts as a substrate to enhance glycosylation of αDG, helping to correct the deficiencies caused by FKRP mutation.^128^

In the past decade, several innovative approaches have been explored that have the potential to enhance or even replace traditional gene therapy methods. CRISPR, for instance, emerged as a method to precisely correct defective genes. Although this technology has not yet reached clinical application for any muscular dystrophy, it was successfully tested *in vitro* for LGMD. Two separate groups have demonstrated effective targeting and correction of the dysferlin, α-sarcoglycan,^129^ and calpain genes.^130^ Others are now focusing on non-viral gene delivery systems, including lipids, polymers, cell-penetrating peptides, and other organic or inorganic materials. In LGMD in particular, Guha et al. delivered plasmid DNA containing the gene of interest in mouse models of LGMDs (R1/2A, R2/2B, R3/2D) isolated or in combination with follistatin (a myostatin inhibitor).^131^

### X-linked myotubular myopathy

X-linked myotubular myopathy (XLMTM) is a rare, genetic neuromuscular disorder caused by mutations in the gene encoding myotubularin (*MTM1*), a phosphoinositide phosphatase that contributes to skeletal muscle development and excitation-contraction coupling.^132^^,^^133^ The clinical presentation of XLMTM commonly includes severe hypotonia, skeletal muscle weakness, and ventilatory insufficiency, often presenting shortly after birth. Historically, most newborns with XLMTM require respiratory support, nearly half of patients do not survive past 18 months of age, and chronic ventilator dependence and tracheostomy is typical of surviving children.^134^^,^^135^

Critical studies in Mtm1-deficient mouse and canine models recapitulated many features of human XLMTM and formed the basis for clinical studies of gene replacement therapy. Using an rAAV8-desmin-Mtm1 product, Buj-Bello demonstrated alleviation of the disease phenotype in the mouse model.^136^ Subsequently, intravenous delivery of canine MTM1 (cMTM1) with progressively escalating quantities of the rAAV8-desmin-cMTM1 vector rescued declining motor and respiratory function in affected dogs.^137^ Treated dogs achieved correction of muscle pathology, and in the long term, retained similar respiratory and neurological function as unaffected control littermates.^138^ The proof-of-concept data in animals demonstrated the potential that MTM1 gene delivery could be feasible, safe, and efficacious for human XLMTM.

The ASPIRO trial (NCT03199469) is a multinational, open-label, dose-escalation phase 1/2 trial assessing the safety and efficacy of intravenous AAV8-Desmin-MTM1 gene therapy (AT132, resamirigene bilparvovec, Astellas Pharma, Inc). The study was originally planned in two parts: the first part involving safety and dose-escalation goals encompassing a lower dose (1.3 × 10^14^ vg/kg), higher dose (3.5 × 10^14^ vg/kg), and a delayed treatment control group. The goal of part two was dose selection and verification. Treated enrollees were also compared with children enrolled in a concurrent natural history run-in study (INCEPTUS, NCT02704273).

Results through February 2022 were published from the 26 participants who enrolled in ASPIRO,^139^ as well as 12 run-in study participants. After allocation of seven participants originally assigned to delayed treatment, ultimately 17 children received the higher dose, seven received the lower dose, and 14 were in the untreated group of control and run-in participants. The initial immunosuppression regimen (prednisone, 1 mg/kg daily) for 8 weeks (4 weeks, followed by a 4-week taper) was extended to 16 weeks (8 weeks, followed by 8-week taper) midway through the study due to observed transaminase (*n* = 1) and troponin-I (*n* = 1) elevations that occurred 7 weeks after dosing. The primary efficacy outcome was change in hours of daily mechanical ventilation support at 24 and 48 weeks post dosing.

At dosing, children were a median of 12 (IQR: 10–30) months of age in the lower dose group, 31 (IQR: 16–64) months in the higher dose group, and 18 (IQR: 10–31) months in the delayed treatment group. The follow-up lasted 46 (lower dose, IQR: 41–49), 27 (higher dose, IQR: 24–29), and 28 (control, IQR: 9–46) months. Twenty-four weeks after receiving AT132, both dosages led to significant reductions in mechanical ventilation use (lower dose: 77% drop, 95% CI: 40–115, *p* = 0.0002; higher dose: 22% drop, 95% CI: 6–39, *p* = 0.0077). Other secondary study outcomes indicated significant respiratory strengthening and achievement of independent sitting in the majority of dosed children.^139^ Since chronic ventilator dependence from XLMTM and other severe congenital myopathies does not naturally recover,^134^^,^^135^ liberation from mechanical ventilation was a major departure from the typical clinical trajectory. No guidance existed for systematically weaning AT132-treated children from mechanical ventilation, so an international group of experts in respiratory care and XLTMTM clinical management was convened to create an algorithm for evaluating weaning readiness and monitoring tolerance to reduction (or removal) of support in trial participants. Principles established by this consensus document can potentially guide ventilator weaning following disease-modifying therapies for other severe myopathies.^140^

Skeletal muscle biopsies were assessed from the first 10 dosed participants, and further subdivided according to the AT132 dose and immunosuppression regimen.^141^ Muscle samples at baseline showed hypotrophic fibers characteristic of XLMTM with increased internal nucleation and presence of organelle aggregates. In follow-up biopsies, organelle localization improved by 24 weeks and myofiber size increased by 48 weeks. Cellular infiltration and more extensive inflammation were observed in participants who received less extensive immunosuppression. When muscle samples from two participants in the higher dose group exhibited more widespread histological recovery, these findings contributed to the selection of the higher dose for part two/dose verification of the trial.^141^

Further transcriptomic analysis from a subset of muscle samples (*n* = 14) revealed dose-dependent transduction and myotubularin protein expression.^142^ Using RNA-sequencing and machine learning approaches, differential upregulation of lipid metabolism and inflammatory response processes was detected by week 48, with downregulation of processes relating to cell-cell adhesion, extracellular matrix organization, and muscle tissue development. Differential expression of myogenesis and interferon pathway genes at 48 weeks was associated with liberation from mechanical ventilation.^142^

Promising interim data released by the sponsor in 2018 revealed clinically meaningful improvements in independent breathing ability and motor function, as compared with untreated controls and historical data.^143^ This led to FDA designation of AT132 as a regenerative medicine advanced therapy (RMAT). Major setbacks began in 2020, when the sponsor reported deaths of three children after receiving the higher dose. Following a 7-month FDA hold and safety review, the study was cleared to resume recruitment using the lower AT132 dose (1.3 × 10^14^ vg/kg). However, the death of a fourth participant in September 2021 triggered a second, ongoing FDA clinical hold. Through February 2022, four children (lower dose *n* = 1; higher dose *n* = 3) experienced fatal cholestatic liver failure following dosing, whereas three children in the untreated control group died (hepatic peliosis, *n* = 1; aspiration pneumonia, *n* = 1; acute on chronic bronchopneumonia, *n* = 1). Additional serious, non-fatal treatment-related hepatobiliary adverse events were reported in five participants (*n* = 4 in the higher dose). A recent report demonstrated loss of Mtm1 in a zebrafish model was associated with severe cholestatic liver disease.^144^ Although the primary study completion and results were released in October 2024,^145^ data collection on longer-term safety and functional endpoints continues.

In June 2023, Astellas announced a license agreement with Kate Therapeutics for KT430, a preclinical *MTM1* gene delivery product that uses an engineered MyoAAV capsid^146^ to de-target the liver. If further understanding and resolution can be achieved for the troubling safety issues uncovered by ASPIRO, the striking improvements in independent breathing and motor function in many dosed participants suggests the potential for meaningful gains.

### Congenital coagulation disorders: Hemophilia

Although there are preclinical data for multiple congenital disorders of hemostasis and thrombosis,^147^ including among others, von Willebrand disease,^148^ factor VII deficiency,^149^ and protein C deficiency,^150^ clinical AAV efforts for coagulation disorders have thus far exclusively targeted hemophilia A (HA) and B (HB). Progress in hemophilia gene therapy now encompasses licensed HA^151^^,^^152^^,^^153^^,^^154^ and HB^15^^,^^155^^,^^156^^,^^157^ vectors as well as a defined series of questions to address, particularly for HA, to improve next generation efforts.^7^^,^^158^^,^^159^ Most hemophilia gene therapy efforts employ intravenously delivered AAV vectors. Outside the scope of this review, lentiviral vectors have shown early clinical trial success for *ex vivo* transduction of hematopoietic stem cells to express human factor VIII (FVIII)^160^ or a human-porcine hybrid FVIII variant^161^^,^^162^^,^^163^ as well as proof-of-concept efficacy in an HA large animal model following intravenous lentiviral vector administration.^164^

HA and HB are the most common X-linked recessive severe congenital bleeding disorders caused by a deficiency in coagulation FVIII or factor IX (FIX) function, respectively.^165^ Current AAV efforts target hepatocyte transgene expression where FIX, but not FVIII, is endogenously expressed.^166^ Factor VIII is primarily expressed in liver sinusoidal endothelial cells.^167^^,^^168^ Hemophilia phenotype correlates with FVIII/FIX plasma levels such that frequent spontaneous bleeding is observed in patients with severe hemophilia (<1% of normal), while bleeding in moderate hemophilia (1%–5% of normal) occurs after minor trauma or occasionally spontaneously and mild hemophilia patients (5%–40% of normal) typically bleed following trauma.^169^ Current standards of care consist of prophylactic intravenous protein replacement or subcutaneous monoclonal antibody administration to prevent bleeding with the goal of converting the severe phenotype to mild hemophilia.^170^ While effective, current therapies have a nearly 40% non-compliance rate, cost ≥200,000 USD annually, have not demonstrated ability to prevent progression of arthropathy and are unavailable to approximately 75% of the world’s hemophilia population.^171^^,^^172^^,^^173^^,^^174^^,^^175^ These unmet clinical needs, combined with well-established animal models, a plasma biomarker that correlates with phenotype, a strong molecular understanding of disease pathology, and well-organized patient and provider communities have made the hemophilias model monogenic disorders for the development of gene-based therapies.

#### Hemophilia B

After initial efforts using AAV vectors for HB^10^ outlined important challenges to overcome, the first report of successful sustained FIX transgene expression was published in 2011.^16^ Currently, all active clinical HB efforts, including the two licensed products (Table 1) incorporate a naturally occurring gain-of-function FIX variant, FIX-Padua or FIX-R338L (Table 2). The FIX-R338L protein has an 8-fold improvement in specific activity^176^^,^^177^ by one-stage clotting assay and improved function, though, to date, the degree of enhanced FIX-R338L *in vivo* hemostatic function over FIX-WT has not been confirmed. There are now two FDA-approved AAV vectors encoding FIX-R338L for HB: etranacogene dezaparvovec (Hemgenix, CSL Behring),^156^^,^^178^ approved in 2022, and fidanacogene elaparvovec (BEQVEZ, Pfizer),^15^^,^^155^ approved in 2024. Etranacogene dezaparvovec is an AAV5 vector administered at a dose of 2 × 10^13^ vg/kg. Fidanacogene elaparvovec is packaged in an rh74-variant capsid and administered at a dose of 5 × 10^11^ vg/kg. The development of Hemgenix was recently comprehensively reviewed.^157^

**Table 2:** AAV gene addition trials for hemophilia A and B

Indication	Sponsor	Dose (× 10^11^ vg/kg)	Transgene	Serotype	Status	NCT
HB	Belief Bio	50	FIX-Padua	clade A derived	Phase 3	NCT05203679
	Pfizer	5	FIX-Padua	rh74var	FDA approved	NCT03587116NCT03861273NCT05568719
	CSL Behring/Hemgenix	200	FIX-Padua	AAV5	FDA approved	NCT03569891
HA	BioMarin/Roctavian	600	FVIII-SQ	AAV5	FDA approved	NCT03370913
	Sangamo/Pfizer	300	FVIII-SQ	AAV6	phase 3 completed^a^	NCT04370054
	Spark	5–20	FVIII-QQ	LK03	phase 2b planned	IND pending
	ASC Bio	20^+^	FVIII-ET3	AAV8	phase 1/2	NCT04676048
	UCL/St. Jude	6–40	FVIII-V3	AAV8	phase 1/2	NCT03001830
	Undisclosed sponsor in China	20–60	FVIII-V3	AAV8	phase 1/2	international trial

a Pfizer opted not to file a biologics licensing application.

Although the Etranacogene dezaparvovec dose is 40-fold higher than fidanacogene elaparvovec, the clinical outcomes of both vectors were comparable in a pivotal trial.^155^^,^^156^^,^^179^ This includes achieving average one-stage FIX activity well into the range of mild HA, similar annualized bleeding rates (1.9, etranacogene dezaparvovec and 1.3, fidanacogene elaparvovec) in which 65%–70% of participants had bleeding rates of 0 during the first year after vector administration. Importantly, 13% (*n* = 6 of 54) of fidanacogene elaparvovec and 6% (*n* = 3 of 45) of etranacogene dezaparvovec patients returned to prophylaxis. All etranacogene dezaparvovec participants who returned to prophylaxis (*n* = 3) had undetectable expression.^156^ In contrast, one fidanacogene elaparvovec participant lost detectable transgene expression within a year post vector. The remaining five fidanacogene elaparvovec trial participants who returned to prophylaxis had FIX activity in the range of moderate or mild HB but resumed prophylaxis electively or after break-through bleeding.^155^ These observations highlight variations in clinical practice as well as inter-patient variability in required factor activity to prevent bleeding. While neither vector presented major safety concerns, approximately two-thirds of fidanacogene elaparvovec participants were treated with glucocorticoids due to a presumed anti-AAV capsid immune response,^155^ compared with 17% of etranacogene dezaparvovec participants.^156^^,^^179^ Similarly, neither vector has confirmed long-term toxicities; however, 20% of Hemgenix participants had hepatic transaminase elevations ≥2 years post vector administration of unclear etiology^157^^,^^179^ that, to date, have not been reported with fidanacogene elaparvovec.^15^^,^^155^

**Figure 2:**
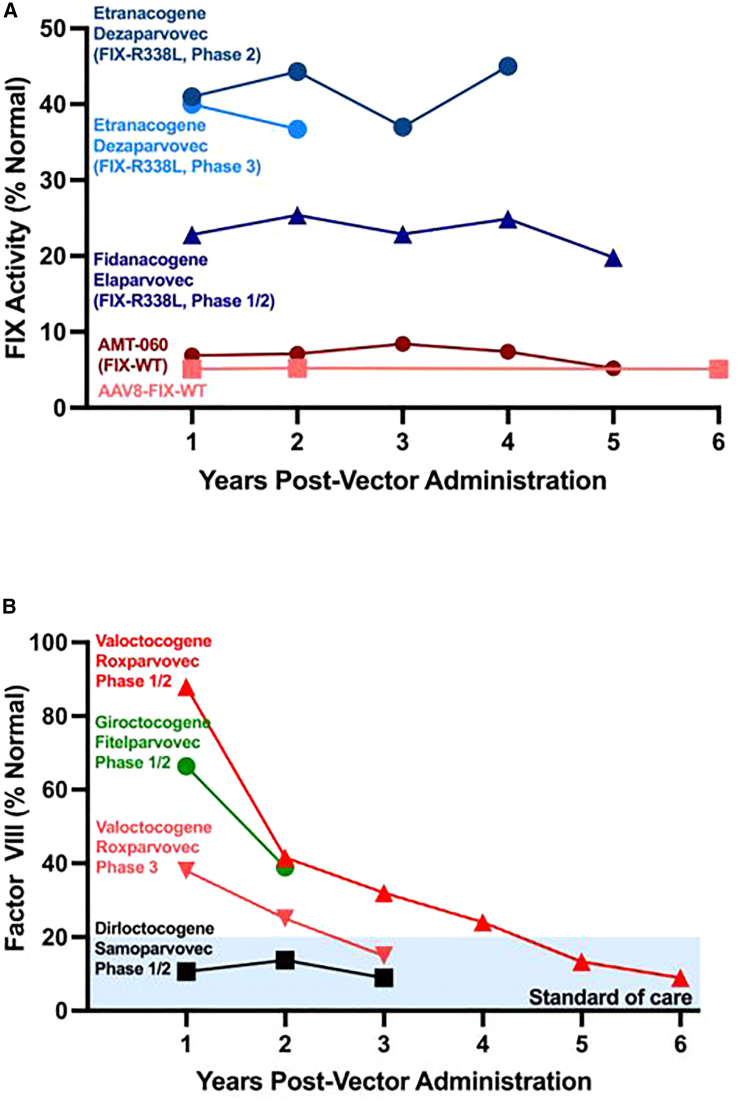
Longitudinal factor activity following AAV gene therapy (A) Mean reported one-stage assay determined factor IX activity of etranacogene dezaparvovec in phase 2^180^ or 3,^181^ fidanacogene elaparvovec phase 1/2,^182^ and AMT-060^180^ and AAV8-FIX-WT phase 1/2 studies.^183^ (B) Factor VIII (FVIII) activity values are median one-stage assay values for dirloctocogene damoparvovec^185^ or calculated one-stage assay as a function of reported chromogenic FVIII activity multiplied by 1.6 are reported for valactocogene roxaparvovec.^179^ Giroctocogene fitelparvovec values^186^ are means as medians values are not reported. The gray shaded area denotes FVIII or FVIII-equivalency obtained by current phylactic standard of care.

Unique from all intravenously administered AAV vectors, etranacogene dezaparvovec eligibility is independent of anti-AAV neutralizing antibodies (NAbs) status.^156^^,^^179^ In contrast, fidanacogene elaparvovec requires undetectable NAb and resulted in the ineligibility of nearly 60% of screened pivotal trial participants.^155^ Importantly, though preexisting NAb do not preclude etranacogene dezaparvovec administration per the label, participants with AAV NAb >1:300 had lower transgene expression than those with titers <1:300 and the single participant with an NAb >1:3,000 did not have expression.^156^^,^^179^ The manufacturer of etranacogene dezaparvovec has committed to a post-marketing study enrolling positive NAb participants to further delineate efficacy in Nab-positive patients.^179^ While unclear, etranacogene dezaparvovec observations may be explained, at least in part, by biologically distinguishing features of AAV5, NAb assay methods, variability of capsid-specific transduction efficiency, and the ability of high AAV vector doses to overcome a, as yet undetermined, threshold of AAV NAb that would need to be carefully considered relative to dose-dependent AAV vector toxicities. Last, early phase clinical trial data from etranacogene dezaparvovec,^180^^,^^181^ fidanacogene elaparvovec,^182^ and prior HB clinical trials^183^^,^^184^ have outlined multi-year stable FIX/FIX-R338L expression (Figure 2A), which differs from durability observations of some HA vectors (Figure 2B).^151^^,^^153^^,^^154^^,^^186^^,^^187^

Ongoing and near-future next generation approaches to HB gene therapy include an *F9* gene integration approach using lipid nanoparticle delivery of *Cas9* and an AAV8-FIX vector.^188^ Long term, it is notable that neonates and young hemophilia pediatric patients are not currently eligible for licensed or investigational therapies and stand to have the greatest lifelong benefit. This highlights the future role of gene editing and/or the ability to re-administer AAV vectors.

#### Hemophilia A

Unlike FIX, FVIII cDNA (7kb) exceeds the 4.7-kb AAV packaging capacity. This requires the use of a truncated FVIII variant where the B-domain is replaced by a peptide linker to maintain FVIII heterodimer formation, most commonly FVIII-SQ (4.4 kb).^189^ The B-domain comprises 40% of the FVIII protein and is removed following activation. While the B-domain has no known procoagulant function, it is thought to contribute to FVIII secretion.^190^ As such, additional engineered B-domain deleted variants have incorporated N-linked glycosylation sites into the B-domain replacing peptide linker, e.g., FVIII-V3,^191^ that are thought to improve secretion, albeit not yet directly demonstrated. This approach^192^ has thus far not translated into multi-year durable FVIII expression beyond the range of mild HA, which has also been observed in at least four trials encoding FVIII-SQ.^185^^,^^186^^,^^193^

There are several ongoing HA clinical trials (Table 2) and a single licensed vector, valoctocogene roxaparvovec (“Roctavian,” Biomarin; Table 1), whose clinical development was recently comprehensively reviewed.^154^ Commercial uptake of valoctocogene roxaparvovec has been anemic, at least in part, due to concerns of expression durability, variability in clinical response, and unclear sustained improvement over current standards of care (Figure 2B).^171^^,^^194^^,^^195^ Although a defined threshold of therapeutic FVIII activity is not established, epidemiologic studies demonstrate that FVIII activity of >12%–20% protects against spontaneous joint bleeds.^196^^,^^197^ This suggests that durable FVIII activity ≥20% is a minimal therapeutic threshold for HA gene therapy to exceed current standards of care and eliminate/nearly eliminate joint bleeds.

Valoctocogene roxaparvovec is an AAV5 vector encoding FVIII-SQ that is administered at a dose of 6 × 10^13^ vg/kg.^151^^,^^153^^,^^198^ In pivotal trial, median 1 one-stage FVIII activity was 40% of normal (range: 0–311) resulting in an impressive 84% reduction in annualized bleeding.^152^^,^^153^ However, like phase 1/2 participants,^187^ pivotal trial data have thus far demonstrated year-over-year loss of FVIII expression (Figure 2B)^151^^,^^153^^,^^154^ with corresponding declines in efficacy.^151^^,^^152^^,^^153^ These observations highlighted transgene expression durability as a major outstanding question in HA gene transfer. Similar loss of FVIII expression was reported in the high-dose cohort (3 × 10^13^ vg/kg) of phase 1/2 participants following giroctocogene fitelparvovec (Pfizer), an AAV6 vector also encoding FVIII-SQ.^186^ This dose of giroctocogene fitelparvovec was pursued in pivotal trial. Interestingly, 50% of phase 3 giroctocogene fitelparvovec participants had supratherapeutic expression at year 1, demonstrating the highest peak FVIII values thus far reported for hemophilia A gene therapy and requiring anticoagulation management.^199^ Interestingly, despite meeting pivotal trial endpoints, Pfizer recently announced they will not file a biologics licensing application and instead return giroctocogene fitelparvovec program back to the original developer (Sangamo Therapeutics), raising uncertainties around the future commercialization of the product.^200^

Thus far, HA vectors (valoctocogene roxaparvovec and giroctocogene fitelparvovec) administered at doses that initially achieved an average of normal/near-normal FVIII activity presented similar declines in FVIII expression.^154^^,^^186^ In contrast, at least four trials (NCT03588299, NCT03003533, NCT03061201, and NCT03001830) demonstrated that initial steady-state FVIII expression in the range of moderate or mild HA was stable on available follow-up data at 2–5 years post vector infusion.^185^^,^^186^^,^^192^^,^^201^ This is consistent with canine HA models that demonstrated no decline in FVIII expression in the range of moderate or mild HA for up to 10 years post vector.^202^^,^^203^ We additionally recently reported HA mice demonstrated FVIII expression level-dependent durability, such that initially high FVIII expression declined by approximately half by 1.5 years post vector administration while lower levels were stable over time.^204^ Thus far, there is not a mechanistic explanation for these observations. Data in mice demonstrated that baculovirus-manufactured vector lost transgene expression due to gene silencing and loss of vector copy number, while mammalian manufactured vector^205^ implicated only loss of vector copy number with evidence of an unfolded protein response.^204^ Importantly, direct comparison of these animal studies and clinical trials is limited by, among other possibilities, differing capsid, expression cassette, manufacturing platforms, and vector dose. Nonetheless, a consistent observation across multiple clinical HA trials and animal models using AAV vectors targeting hepatocyte expression is that FVIII levels in the range of moderate or mild HA is durable, while higher levels of FVIII expression are not. This suggests the mechanism of FVIII expression durability is, at least in part, related to the amount of FVIII expressed. In turn, this may provide a strong rationale to employ the lessons learned by adapting FIX-Padua for HB and incorporate an enhanced potency FVIII variant. The rationale being an enhanced function FVIII variant may normalize FVIII hemostatic function at low levels of expression that permit durability and can be achieved at low AAV vector doses to minimize toxicities. Multiple bioengineered FVIII variants with improved function or secretion have been described.^206^^,^^207^^,^^208^^,^^209^ The first planned clinical trial incorporating an enhanced function FVIII variant, FVIII-R336Q,R562Q or FVIII-QQ,^207^ was recently announced by Spark Therapeutics and Roche.^210^

### Cardiac indications of intravenous AAV gene therapy

Initial studies have used intravenous AAV administration to target the heart, aimed to restore normal levels of the cardiac isoform of SERCA2a, a protein critical for cardiomyocyte calcium regulation and hypothesized to be necessary to improve cardiac function in heart failure.^211^^,^^212^^,^^213^^,^^214^^,^^215^ While early trials using direct intra-coronary artery AAV administration showed encouraging improvements in cardiac function and symptoms, the larger CUPID phase 2b trial ultimately failed to meet its primary and secondary efficacy endpoints.^213^ The lack of efficacy was attributed to inefficient gene delivery to the target tissue, prompting iterative next generation ongoing efforts using higher intravenous AAV vectors doses (3 × 10^13^ to 6 × 10^13^ vg/kg) that aim to improve vector target cell transduction and therapeutic efficacy (NCT04703842 and NCT05598333). Though the prior CUPID trials were not efficacious, they were crucial in demonstrating that AAV vectors can safely transduce cardiac tissue, and highlighted the need for more efficient, cardiotropic vectors with enhanced transduction efficiency. Building on these early efforts, additional groups have expanded cardiac-directed AAV gene therapy to target monogenic disorders that result in cardiomyopathy due to primary metabolic disorders (e.g., Danon disease [DD]), disorders of desmosomal proteins (e.g., arrhythmogenic cardiomyopathy), sarcomeric proteins (e.g., hypertrophic cardiomyopathy) or mitochondrial disorders (e.g., Friedreich’s ataxia). In this section, we discuss ongoing clinical trials using intravenous AAV vectors for monogenic disorders resulting in cardiomyopathy.

#### Danon disease

DD is a rare, X-linked lysosomal storage disorder caused by loss-of-function mutations in the *LAMP2* gene, which encodes lysosome-associated membrane protein type 2.^216^ This condition affects multiple organ systems, including the central nervous, skeletal, and cardiovascular systems, the latter being the dominant cause of morbidity and mortality due to rapidly progressive hypertrophic cardiomyopathy. Males with this disorder experience severe arrhythmias and eventual heart failure, with a median survival of 19 years without heart transplantation.^217^^,^^218^ Females with a pathologic genotype have a highly variable clinical phenotype and significantly better survival than males.

The LAMP2 gene produces three alternatively spliced isoforms: LAMP2A, LAMP2B, and LAMP2C, each playing distinct roles in autophagy. Deficiency of LAMP2B, the predominant form expressed in cardiomyocytes, causes mitochondrial and contractile abnormalities in the heart.^219^ The therapeutic potential of LAMP2B restoration therapy for DD has been demonstrated in patient-derived iPSC-CMs as well as in LAMP2 knockout mice, showing reduced accumulation of autophagic vacuoles and improved cardiac function.^220^

Based on experimental success, Rocket Pharmaceuticals initiated the first-in-human gene therapy trial for a monogenic cardiomyopathy in 2019. The open-label phase 1 clinical trial (NCT03882437) evaluated RP-A501 (AAV9.LAMP2B), administered by intravenous infusion to adult and pediatric male patients with DD across two dose cohorts (6.7 × 10^13^ vg/kg and 1.1 × 10^14^ vg/kg). One of the two adult patients treated with the higher dose developed complement-mediated thrombotic microangiopathy (TMA) with renal impairment, leading to the suspension of further high-dose enrollment and adjustments to the immunomodulatory regimen.^221^ Despite this, all serious adverse events (SAEs), such as platelet reduction, transaminase elevation, and steroid-induced myopathy, were manageable and fully reversible. Preliminary evidence of efficacy was reported in both adult and pediatric patients, with sustained LAMP2 expression and decreased vacuolar area in endomyocardial biopsies.^222^ Additionally, cardiac structure and function were improved or stabilized over a follow-up period of up to 24 months. Biomarkers of cardiac injury and disease, including brain natriuretic peptide (BNP) and high-sensitivity troponin-I (hsTnI), were also reduced. Overall, RP-A501 was tolerated across pediatric and adult cohorts and a single-arm, multicenter phase 2 clinical trial (NCT06092034) aiming to further evaluate efficacy of the lower dose is now under way^223^ and the results of 5-year outcomes from the phase 1 study were recently published.^224^

#### Arrhythmogenic cardiomyopathy

Arrhythmogenic cardiomyopathy (ACM) is an autosomal dominant genetic disease caused by loss-of-function mutations in genes that encode desmosomal proteins. Pathologically, ACM is characterized by chamber dilation and fibro-fatty replacement of the myocardium, which constitutes a substrate for electrical instability and life-threatening ventricular arrhythmias.^225^^,^^226^ The most commonly affected gene is *PKP2*, which accounts for 20%–45% of all ACM cases.^227^ *PKP2* disease-causing mutations lead to disrupted cell-cell junctions and electrical signaling beyond the desmosome, eliciting fibrosis and inflammation in the heart.^228^^,^^229^ Due to limited therapeutic interventions, ACM has a poor prognosis, constituting a leading cause of sudden cardiac death (SCD) most notably in pediatric patients.

Over the past year, four different groups have reported the efficacy of AAV-mediated PKP2 overexpression to mitigate disease progression, improve cardiac function, and extend lifespan in various mouse models of PKP2-associated ACM.^230^^,^^231^^,^^232^^,^^233^ Based on these studies, three investigational new drugs have been approved by the FDA to advance into phase 1 clinical trials. These open-label, dose-escalating trials will evaluate safety and preliminary efficacy of a single AAV-mediated delivery of *PKP2* into adult patients with implantable cardiac defibrillators (ICD) (NCT06228924, NCT05885412, NCT06109181). All these gene therapy products carry a functional copy of the human *PKP2* gene but differ on their selected AAV capsids. TN-401 (Tenaya Therapeutics) uses AAV9, while LX2020 (Lexeo therapeutics) and RP-A601 (Rocket Pharmaceuticals) use AAVrh10 and AAVrh74, respectively. Dosing has or will soon be initiated at 2 to 8 × 10^13^ vg/kg, variable among the different studies.

#### Hypertrophic cardiomyopathy

Cardiac myosin-binding protein C (MyBP-C) is a structural protein of the sarcomere that interacts with actomyosin filaments to modulate cardiac contractility.^234^^,^^235^ Reduced levels of this protein due to loss-of-function mutations in the MYBPC3 gene, are a leading cause of hypertrophic cardiomyopathy (HCM).^236^ The clinical presentation of MYBPC3-HCM includes adverse cardiac remodeling and diastolic dysfunction that often progress to heart failure and arrhythmias.^236^ Intravenous AAV9 administration to deliver an optimized MYBPC3 gene (TN-201) to the heart is currently under investigation in a phase 1 multicenter, open-label trial (NCT05836259) sponsored by Tenaya Therapeutics. This study aims to evaluate the safety, tolerability, and early clinical efficacy of a single intravenous infusion in patients with nonobstructive MYBPC3-HCM who have an ICD.^237^ The first dose of TN-201 being assessed is 3 × 10^13^ vg/kg, a dose associated with restoration of MYBPC3 protein levels and improved cardiac function in a preclinical mouse study preprint shared by the company.^238^

#### Friedreich’s ataxia

Friedreich’s ataxia (FRDA) is a rare, inherited autosomal recessive disorder that causes progressive difficulties with balance, coordination, and speech leading to loss of ambulation and early mortality. It is caused by a biallelic expansion of a GAA repeat in the *FXN* gene, leading to impaired production of frataxin, critical for mitochondrial activity. As the disease progresses, neurological symptoms including difficulty walking and poor balance, impaired sensory functions and muscle coordination, loss of reflexes, and slurring of speech, worsen over time. Additonally, cardiomyopathy, arrythmia and cardiac fibrosis are freqently observed in FRDA patients, and are responsible for a significant proportion of deaths.

Currently, the only FDA-approved therapy for the treatment of FRDA is omaveloxolone (SKYCLARYS), a semi-synthetic titerpenoid small molecule that imparts therapeutic function through activation of the Nfr2 pathway, which is impaired in FRDA patients leading to oxidative stress and mitochondrial impairment.^239^ Omaveloxolone was approved based on a significant, albeit modest, effect on the neurological progression rate but its effect on cardiomyopathy was not assessed. There is a need for more effective strategies to improve neurological outcomes and address all the manifestations of FRDA, including cardiomyopathy.

Delivery of *FXN* gene by AAV-mediated gene therapy has demonstrated improvements in cardiac and neurological function in preclinical models.^240^^,^^241^^,^^242^^,^^243^^,^^244^ Importantly, several studies have also noted the potential for toxicity from overexpression of frataxin. Initiation of a gene replacement strategy for the neurological targets involved in FRDA will require development of appropriate serotypes and/or optimized delivery routes to enable efficient transduction of cellular CNS targets. Further development of strategies is needed to target both the CNS and other systemic organs affected, such as the heart, pancreas, and skeletal muscle, as well as deficits in vision and hearing. Other general issues related to gene therapy include minimizing the AAV-associated toxicities and providing the gene product under appropriate regulatory control to achieve required levels of frataxin expression while avoiding overexpression.

There is currently a clinical trial evaluating the safety and efficacy of *FXN* gene replacement therapies that target cardiomyopathy in FRDA patients. The rationale for this trial is based on the knowledge that cardiomyopathy is an important cause of mortality and that lower doses delivered intravenously can achieve this with lesser potential for adverse events. Lexeo Therapeutics recently announced interim data of LX2006, an AAV-based gene therapy delivered intravenously, for the treatment of FRDA cardiomyopathy. LX2006 can transfer the *FXN* gene to myocardial cells and consequently increase levels of frataxin in the mitochondria, restoring mitochondrial function in cardiac myocytes. Preliminary data support that LX2006 was well tolerated across both the Lexeo SUNRISE-FA phase 1/2 clinical trial (NCT05445323) and the Weill Cornell Medicine Phase 1A trial (NCT05302271), with no treatment-related serious adverse events and no signs of complement activation or other types of immunogenicity. Additionally, clinically meaningful improvements in cardiac biomarkers were observed.^245^^,^^246^^,^^247^ Though these preliminary data are promising, these cardiac-directed trials must be viewed in the context of incomplete natural history of FRDA-associated cardiomyopathy, and the likely inability of these patients to receive repeat AAV vector infusion in the event of future AAV delivery of frataxin to the CNS becomes available.

Important developments in the directed evolution of cardiac-targeted AAV capsids should accelerate the clinical development of additional products for inherited cardiomyopathy. A variety of ligands have been successfully added to VR-VIII of the AAV9 capsid to increase cardiac tropism as much as 10-fold over the parental vector. In addition, multi-species evolution of cardiac-targeted capsids should facilitate the further development of cardiotropic capsids, especially when all surface epitopes are subjected to directed evolution. Since clinical and imaging outcomes are well established in inherited cardiomyopathy, it should be possible to see an increase in the number of effective products for inherited heart disease.

### AAV for lysosomal storage disorders

Lysosomal storage disorders (LSDs) are a diverse group of >70 autosomal recessive or X-linked genetic disorders caused by defects in lysosomal enzymes, transporters, co-factors, or related proteins, leading to the accumulation of undigested substrates within the lysosome. These mutations result in a range of clinical manifestations, which can vary in severity and age of onset depending on the specific disorder and the function of the affected protein. Common features include organomegaly (e.g., hepatosplenomegaly), neurological decline, skeletal deformities, and other systemic complications. LSDs often affect multiple organs and systems, leading to progressive disability if left untreated.^248^ In addition to trials of intravenous delivery of AAV vectors for LSD outlined herein, clinical trials have been conducted for Gaucher disease and neuronal ceroid lipofuscinoses,^249^^,^^250^ the latter pursued by direct central nervous system delivery. Despite known efficacy endpoints and an established regulatory pathway established by enzyme replacement therapies for select LSDs, AAV gene therapy progress has been modest due to, among other challenges, CNS delivery and the unknown relative percentage of cells necessary to express the transgene for clinical benefit. There is an opportiuntiy in LSD gene therapy to seek accelerated approval based on normalization of the abnormal accumulation of substrate in target tissues, especially the brain, where conventional enzyme replacement therapy has been of limited success.

#### Fabry disease

Fabry disease (FD) is a rare genetic disorder caused by mutations in *GLA* gene, which leads to a deficiency of the enzyme α-galactosidase A.^251^ This deficiency results in the accumulation of glycosphingolipids in various tissues, causing progressive damage to the kidneys, heart, skin, and nervous system. While enzyme replacement therapy (ERT) remains the primary treatment since 2001, its requirement for lifelong biweekly infusions makes single-infusion AAV gene therapy an appealing alternative.^252^

Sangamo Therapeutics’ ST-920 uses an AAV2/6 capsid to deliver a human GLA transgene to liver cells. Interim results from the phase 1/2 STAAR trial (NCT04046224) have shown sustained increases in α-Gal A levels for up to 3 years after ST-920 infusion, with patients reporting improved symptoms and quality of life.^253^ Notably, most participants were able to discontinue ERT. A similar approach is being pursued by uniQure, which has recently opened enrollment for a phase 1/2 clinical trial in patients with suboptimal response to ERT (NCT06270316).

To target cardiac manifestations in FD, 4D Molecular Therapeutics is developing 4D-310, which employs a cardiac-directed capsid (C102) carrying a GLA transgene under a ubiquitous promoter. Early data from phase 1/2 trials (NCT04519749, NCT05629559) have indicated GLA expression in cardiomyocytes, along with improved cardiac function and exercise capacity.^254^ However, three out of six patients developed transient atypical hemolytic uremic syndrome (aHUS), causing the FDA to place the program on temporary clinical hold. In response, the company initiated a preclinical study in non-human primates to assess the safety and biodistribution of the therapy using a new immunosuppression regimen based on rituximab/sirolimus instead of the previous prednisone regimen. The clinical protocol has also been modified, and the company plans to resume trial development soon.

#### Pompe disease

Pompe disease is an autosomal recessive condition caused by mutations in the acid-α-glucosidase (GAA) gene, an enzyme responsible for lysosomal glycogen degradation.^255^ Pompe disease has an incidence of approximately 1:18,000 births based on newborn screening data,^256^ although historically, based on clinical findings, the incidence ranged between 1:35,000 and 1:138,000 live births.^257^ The disease leads to progressive glycogen accumulation, primarily in cardiac, skeletal, and smooth muscle, as well as in endothelial cells and motor neurons and the central nervous system, leading to cardiac and respiratory dysfunction, progressive neuro-degeneration, vasculopathy, and cognitive impairment.^258^^,^^259^^,^^260^^,^^261^ Pompe disease is traditionally classified as infantile-onset Pompe disease (IOPD) and late-onset Pompe disease (LOPD).^262^ Classical IOPD is characterized by hypotonia, cardiomegaly, and cardiorespiratory failure in the first year of life.^262^ LOPD presents later in life and cardiomyopathy is not evident,^262^^,^^263^ but respiratory insufficiency and progressive muscle weakness are prominent.^264^^,^^265^^,^^266^

Enzyme replacement therapy (ERT) for Pompe disease using recombinant human acid-α-glucosidase (rhGAA) was approved by the FDA in 2006, with newer formulations approved in recent years.^267^^,^^268^ ERT improves survival, cardiac and respiratory disease related to IOPD,^267^^,^^269^^,^^270^^,^^271^^,^^272^ and leads to a modest improvement of walking distance in the 6-min walk test, reduces ventilation time ,and has a small effect on forced vital capacity in patients with LOPD.^273^ However, ERT does not cross the blood-brain barrier, requires biweekly infusions, and its chronic administration can result in immunological reactions toward rhGAA.^274^^,^^275^^,^^276^ Gene therapy for Pompe disease constitutes an attractive approach, as it has the potential to treat skeletal, cardiac, and smooth muscle, as well as central nervous system manifestations potentially with a single dose of the product.^277^ Importantly, for AAV therapies to be most effective in Pompe disease, the primary transduction of CNS will be required. Strategies in which alpha-glucosidase is secreted by liver-directed gene therapy may not be substantially differentiated from ERT. One additional strategy is the use of fusion proteins to alpha-glucosidase which facilitate cross-correction and offer the potential to cross the blood-brain barrier.

AAV vectors are the most studied mechanism to deliver functional GAA in patients with Pompe disease. Multiple AAV serotypes and promoters have been studied in preclinical and clinical settings.^278^^,^^279^^,^^280^^,^^281^^,^^282^^,^^283^^,^^284^^,^^285^^,^^286^^,^^287^^,^^288^ Several clinical trials using AAV vectors have been reported so far. The first-in-human trial using AAV1 was performed by direct injection of rAAV1-CMV-hGAA into the diaphragms of nine pediatric patients with Pompe disease and respiratory insufficiency (NCT00976352).^289^^,^^290^^,^^291^ The product was considered safe and effective at improving ventilatory outcomes in all subjects, primarily by reducing time of assisted breathing and improving tidal volume.^289^^,^^290^ Subjects from this cohort participated in inspiratory strength training, demonstrating benefits particularly in subjects with higher neuromuscular function.^289^ All children received concomitant ERT, and three of them received immunomodulation, which resulted in reduced anti-capsid and anti-transgene antibody response.^289^^,^^290^ Another phase 1/2 clinical trial using AAV9 encoding a codon-optimized GAA and the desmin promoter was performed by Corti et al. (NCT02240407).^292^ The study proposed the administration of immunomodulation with sirolimus and rituximab before the vector injection, followed by a second dose 4 months after the initial dose.^292^

Intravenous administration of AAV-mediated gene therapy through intravenous injection achieves gene transfer to the liver and skeletal and cardiac muscle.^283^^,^^288^ ACTUS-101 is the first intravenous, liver-directed gene therapy trial for patients with LOPD (NCT03535673).^283^ ACTUS-101 used an AAV8 vector and a liver-specific promoter (LSP) at a dose of 1.6 × 10^12^ vg/kg.^293^ The study included three participants, all male, between the ages of 52 and 71. Participants received immune prophylaxis with oral prednisolone. All subjects showed sustained serum GAA activity and discontinued biweekly ERT after week 26.^293^ Muscle biopsy at week 24 revealed unchanged muscle glycogen content in two of three subjects, but muscle GAA activity was increased at week 52.^293^ One subject restarted ERT at week 97.^293^

Another phase 1/2 clinical trial (RESOLUTE) carried by Spark therapeutics used a similar liver-directed approach to deliver SPK-3006, using a proprietary AAVRh74-derived capsid and a codon-optimized secretable GAA cDNA (NCT04093349).^40^^,^^41^^,^^42^ The study has enrolled four subjects but results have not been published to date^43^ and the program is now discontinued.

A more recent open-label phase 1/2 clinical trial (FORTIS), sponsored by Astellas Pharma, used AT845 (AAV8-eMCK-hGAA) in adult patients with LOPD (NCT04164105).^294^ Five participants received a dose of either 3 × 10^13^ vg/kg (*n* = 2) or 6 × 10^13^ vg/kg (*n* = 3).^295^ Results of up to 2 years of follow-up were presented.^295^ Three participants developed transient, steroid-responsive transaminitis, possibly related to AT845.^295^ One participant receiving the higher dose developed peripheral sensory neuropathy and was considered a severe adverse event.^295^ AT845 vector transduction in the muscle and urine glucotetrasaccharide (Glc4) remained stable after withdrawal from ERT in three subjects 1 year post treatment.^295^ Three participants discontinued ERT (at 10, 17, and 24 weeks after dosing), and they remained off of ERT at the 24-month follow-up.^295^

A phase 1/2 clinical trial sponsored by Genecradle Therapeutics using intravenous AAV9-hGAA in infants with IOPD is ongoing (NCT05793307).^296^ Results of one participant were published in a non-peer-reviewed manuscript.^297^ The infant was diagnosed with IOPD and immediately received two doses of ERT prior to receiving GC301 (rAAV9 with a with a codon-optimized human GAA).^297^ The participant received prophylactic prednisolone for a total of 58 days, starting on the day before dosing.^297^ Neuromotor testing showed an improvement from 54 to 69 on the Hammersmith Infant Neurological Examination (HINE) 8 weeks after gene therapy administration (day 56).^297^ In addition, GAA levels increased after dosing and remained elevated 8 weeks after dosing. Cardiac imaging revealed a small improvement of ejection fraction, reduction of the left ventricular mass index, left ventricular weight, and left ventricular posterior wall thickness.^297^ No severe adverse events were reported as part of the trial; however, long-term data are not available.^297^

To achieve adequate transgene expression in the central nervous system, direct intrathecal, intracisternal, spinal, and intracerebro-ventricular administration have been studied in animal models of Pompe disease.^277^^,^^298^^,^^299^ Injection of AAV9-CAG-hGAA or AAVrh10-CAG-hGAA resulted in functional neurologic and cardiac improvement but no changes in muscle glycogen.^298^ Intraspinal administration of AAV5-GAA at the C3-C4 level in Gaa^−/−^ mice to target the phrenic nerve nucleus area decreased intraneuronal glycogen content and improved ventilation, even without enzymatic activity in the diaphragm.^299^

In recent studies, an AAV9 product encoding an excitatory designer receptor exclusively activated by designer drugs (DREADD) (AAV9-hSyn-hM3D(gq)-mCherry) was injected to the posterior tongue of Gaa^−/−^ mice, and demonstrated retrograde movement of AAV9 to hypoglossal motoneurons in all mice, an approach that could address bulbar dysfunction in patients with Pompe disease.^300^

Besides the use of AAV vectors, hematopoietic stem and progenitor cell-mediated lentiviral gene therapy (HSPC-LVGT) is being studied for the treatment of Pompe disease. Hematopoietic stem cell transplantation using GAA-modified HSPCs via lentiviral vectors demonstrated long-term engraftment and continuous supply of GAA after one intervention in Gaa^−/−^ mice, with improvement of motor and cardiac function.^301^ However, the approach required a high vector copy number (VCN) and did not achieve glycogen reduction to normal levels.^301^ Recent studies with this strategy have generated engineered GAA coding sequences, peptide tags, and codon optimization.^302^^,^^303^ A novel vector with a codon-optimized GAA sequence fused to codon-optimized human IGF2 (LV-IGF2.GAAco) lead to correction of glycogen accumulation, autophagy, motor function and brain glycogen content at a much lower VCN.^302^ A similar approach with lentiviral-mediated gene therapy with glycosylation independent lysosomal targeting tags increased secretion and reduced glycogen, myofiber, and CNS vacuolation, but maintained low GAA activity.^303^ Importantly, the use of HSPC-LVGT can limit immunoglobulin G responses, a benefit of this methodology.^304^

#### Mucopolysaccharidoses disorders

Mucopolysaccharidoses (MPS) disorders are a group of progressive, LSDs sharing the common feature of accumulation of glycosoaminoglycans (GAGs) in cells throughout the body. While the MPS subtypes differ in their specific genetic etiology and particular accumulating GAG species, many share clinical features of orthopedic, cardiac, respiratory, and neurologic complications.^305^ Although enzyme replacement therapy (ERT) is the standard of care for many MPS subtypes, none of the currently approved ERTs target the brain.^306^ As such, most gene therapies under development aim to correct the central nervous system (CNS). Both *ex vivo*, lentiviral gene therapy with hematopoietic stem cell transplantation^307^ and *in vivo* AAV-mediated gene replacement strategies are in development for MPS disorders. Regardless of the specific modality, the impact of gene therapy in MPS may be amplified by cross-correction. Through this phenomenon, a small population of genetically corrected cells can secrete the missing functional enzyme to improve the function of neighboring cells.^308^

MPSI (Hurler syndrome) and MPSII (Hunter syndrome) are the most common MPS subtypes,^309^ sharing many clinical and pathophysiologic features. As such, companies have pursued both indications in parallel. Sangamo Therapeutics sponsored a trial of intravenously administered AAV-delivered zinc-finger nucleases, with the goal of editing the albumin locus in liver to express the missing enzyme in each disorder (NCT02702115 and NCT03041324). Hepatic editing levels were low in both studies, resulting in no long-term expression of the missing enzyme in blood.^310^

More recently, REGENEXBIO has developed AAV9-delivered gene replacement therapies administered into the cerebrospinal fluid (CSF) via the cisterna magna for both MPSI (RGX-111, NCT03580083) and MPSII (RGX-121, NCT03566043, NCT04571970). RGX-111 reduced accumulation of GAG species in both CSF and urine.^311^ However, the sponsor announced they are not advancing the program. Conversely, a phase 2/3 trial of RGX-121 was completed. In this trial, MPS II patients experienced a significant reduction in the levels of a key CSF GAG species.^312^ The sponsor has reported plans to file for accelerated FDA approval based on this biomarker improvement.

The MPSIII subtypes (Sanfilippo syndrome types A–D) are phenotypically similar disorders characterized by severe, progressive neurodegeneration. To date, gene therapy trials have been conducted for the two most common subtypes: MPSIIIA and B. A phase 1/2 study of intracerebral injection of AAVrh10-h.SGSH.SUMF1^313^ (LYS- SAF-301, NCT01474343) was found to be safe. In a subsequent phase 2/3 trial, the vector was reengineered to have a stronger CAG promoter, in place of the previous PGK promoter.^314^ While full trial results have not yet been published, the sponsor reported that the trial (AAVrh10-h.SGSH LYS-SAF302, NCT03612869) failed to meet the primary efficacy endpoint. However, subgroup analysis revealed a potential benefit in children dosed at a younger age (<30 months), prior to significant neurologic regression.

A similar age-dependent benefit has been demonstrated in trials of intravenous AAV9-mediated gene replacement in both MPSIIIA (ABO-102, NCT04088734, NCT02716246) and MPSIIIB (ABO-101, NCT03315182). While the MPSIIIB program demonstrated biochemical efficacy, including reduction in CSF heparan sulfate GAG levels,^315^ it was discontinued by the sponsor. Ultragenyx advanced ABO-102 through a phase 2/3 trial, with initial results indicating both a significant reduction in CSF heparan sulfate GAG levels and preservation of neurocognitive development in individuals treated before 30 months of age.^316^ Ultragenyx has reported that they also plan to apply for accelerated FDA approval based on improvements in CSF heparan sulfate levels. This may represent an emerging trend to seek FDA approval via the accelerated pathway, based on biomarker outcomes in these severe, rare disorders.^317^

## CNS-directed gene therapy

While AAV vectors have some ability to cross the blood-brain barrier (BBB), systemic delivery inefficiently targets the brain, particularly deep structures, and spinal cord. Efforts to overcome this limitation include direct delivery methods to CNS tissues (e.g., intracerebro-ventricular injection [ICV], or intraparenchymal injection [IP], or intrathecal injection [IT]) and/or capsid engineering.^3^^,^^240^^,^^318^^,^^319^ Advances in vector engineering and delivery strategies are ongoing to improve AAV’s ability to cross the BBB and effectively deliver gene therapy to the brain and spinal cord. Approaches being pursued include capsid engineering via modifying capsid amino acids and/or incorporating targeted ligands, peptides, or nanoparticles to increase binding and transportation across the BBB. Improving AAV capsids for CNS targeting holds the promise of improving gene therapy by increasing delivery precision, expanding treatable diseases, reducing immune reactions, boosting transduction efficiency, and minimizing off-target effects.

Clinical efforts are ongoing for multiple CNS disorders. This includes gene addition studies for neurodegenerative diseases such as Parkinson’s disease, Alzheimer’s disease, and Huntington’s disease. Additionally, work is ongoing for Tay Sachs disease,^320^ GM1 gangliosidoses, and Rett syndrome. A trial for Rett syndrome sponsored by Neurogene highlighted that high IT AAV9 doses have significant systemic distribution that can result in systemic AAV toxicities, including mortality.^321^ This recent tragic event highlights the need for improved and localized AAV delivery to target CNS tissue that will make AAV-based therapies more effective, accessible, and safe for neurological disorders. Despite these challenges, herein we highlight thus far successful clinical trial efforts for giant axonal neuropathy, Canavan disease, and aromatic l-amino acid decarboxylase deficiency, the latter now a licensed therapeutic.

### Giant axonal neuropathy

Giant axonal neuropathy (GAN) is an ultra-rare autosomal recessive pediatric neurodegenerative disorder manifesting with progressive sensorimotor peripheral, optic, and autonomic neuropathy; central nervous system involvement (including cerebellar dysarthria and dysphagia, variable learning disability, and seizures); and respiratory failure, typically resulting in death in the second to third decade of life.^322^^,^^323^^,^^324^^,^^325^ It is caused by biallelic loss-of-function variants in *GAN*, the gene encoding for gigaxonin, a protein responsible for the regulation of intermediate filament turnover.^322^^,^^323^ Pathological accumulation of intermediate filaments in this disease leads to axonal swellings (giant axons) within the central and peripheral nervous systems. There is a slower progressing Charcot-Marie-Tooth (axonal CMT)-plus disease phenotype, still caused by biallelic *GAN* variants, but with slower decline in function, later loss of electrophysiologic response, and with absent or less CNS disease burden both clinically and by neuroimaging.^324^^,^^325^ Prior preclinical and IND-enabling work demonstrated that intrathecally (IT) administered AAV9 vector can effectively transduce neurons and glia.^326^^,^^327^^,^^328^^,^^329^ In a first-in-human IT gene transfer study, the safety and efficacy of scAAV9/JeT-GAN, a self-complementary AAV-based gene therapy, was evaluated in children with the prototypical phenotype of GAN.^330^ Concurrent immunosuppression with steroids (single-pulse intravenous methylprednisolone with maintenance oral prednisone) was used in all participants, and T cell targeted immunomodulation (rapamycin and tacrolimus) was used for dosing of participants with biallelic null *GAN* variants who were deemed at higher risk for a potential anti-transgene immune response. Fourteen participants received one of four scAAV9/JeT-GAN doses: 3.5 × 10^13^, 1.2 × 10^14^, 1.8 × 10^14^, or 3.5 × 10^14^ total vector genomes (by qPCR titer methodology), with baseline age ranging from 6.3 to 14.5 years (mean age 9.1 years).^330^

The primary study endpoint was safety. During a safety observation period ranging from 8.6 to 90.5 months, 129 adverse events at least possibly related to scAAV9/JeT-GAN and 48 total serious adverse events (SAE) were recorded, with the most common events including elevated cerebrospinal fluid (CSF) white blood cell pleocytosis (lymphocyte predominant), elevation of CSF IgG index, leukocytosis, thrombocytosis, and headache.^330^ CSF pleocytosis occurred in nearly all participants (*n* = 13), peaked by 3–6 months of post gene transfer, was steroid responsive and clinically asymptomatic, and improved or fully resolved by 1 year post gene transfer.^330^ Occurrences of mild and transient grade 1 transaminase elevation were recorded at varying time points (event onset week-3 to year-6 post gene transfer), were often associated with other intercurrent illnesses, and did not require steroid modification or restarting of steroid therapy. Two participants died of disease-related respiratory complications (at 8.5 months and 5 years post gene transfer). One SAE was deemed at least possibly related to an investigational product (due to the temporal proximity from gene transfer) in a participant 2 weeks post gene transfer, who developed fever and emesis and was admitted for intravenous fluid bolus and monitoring. Another infectious etiology was not identified, and the event was associated with CRP and BNP elevation. This event was not associated with troponin elevation, thrombocytopenia, thrombotic microangiopathy, or proteinuria, as can be seen with intravenous gene transfer.^11^^,^^76^^,^^331^ One participant with benign ethnic neutropenia had a transient grade 3 exacerbation of neutropenia due to temporal relationship following gene transfer.

Participants were baseline serum anti-AAV9 neutralizing antibody (NAb) positive (*n* = 6) or negative, but all participants had a negative baseline CSF anti-AAV9 NAb response. Baseline seropositive participants have a higher anti-AAV9 NAb titer at 3 weeks post dosing (range: 1:2,560–1:40,960; median 1:6,400) compared with those who are seronegative at baseline (range: 1:1,280–1:10,240; median 1:2,560). One participant with baseline-positive anti-AAV9 NAb response and the first participant at the 1.8 × 10^14^ v.g. dose cohort developed a marked CSF white blood cell (WBC) elevation to 113/mm^3^ at 3 months post gene transfer, which was clinically silent, responsive to reintroduction of steroids, and was lymphocyte predominant. Subsequent participants received a 4-month oral steroid course post gene transfer, which may have dampened the extent of CSF pleocytosis observed at those dose levels. Anti-AAV9 NAb responses remain elevated in CSF through the final lumbar puncture (12 months) and in blood through at least 7-years post gene transfer. T cell interferon-γ response in peripheral blood mononuclear cells (pBMCs) showed response to capsid-associated peptide pools, but not the transgene (gigaxonin). The use of T cell immune modulation led to lower relative capsid-associated interferon-γ response than those receiving steroids alone. Shedding analysis suggests that more prolonged and higher levels of detection of viral vector in blood, as assessed by quantitative PCR, occurred in individuals who were baseline seronegative, received T cell immune modulation, and/or received higher total vector genome gene transfer dose.^330^

The primary efficacy endpoint was evaluated as change in motor function measure (MFM)-32 total percent score, from baseline through 1 year, compared with pretreatment natural history decline (with estimated slope of −7.17 annual decline).^330^ The mean change in slope of MFM-32 through 1 year post gene transfer improved in the three highest dose groups (+3.23 to +5.32 compared with natural history).^330^ While the minimal clinically important difference (MCID) for GAN has not been established, the relative improvement in MFM-32 rate of change observed in participants with GAN post gene transfer exceeds the MCID for MFM-32 total score that has been reported in other disease: 2.5–3.9 percentage points for collagen VI and LAMA2-related congenital muscular dystrophies and 3 points for spinal muscular atrophy.^332^^,^^333^ In the natural history of disease and classical GAN phenotype, median sensory nerve action potential (SNAP) responses are universally lost in participants by age 9 years and are not recovered once lost. Over long-term follow-up, six participants retained, improved, or regained previously lost median and ulnar SNAP response amplitude, with time to plateau in the decline or reemergence of response ranging from 6 to 24 months post gene transfer.^330^ Of these six participants, the SNAP responses that were improved or regained are still present at last follow-up (age ranging 10–15 years old, and time interval post gene transfer at last follow-up ranging 36–84-month visits post gene transfer). The restoration of upper extremity sensory responses once lost and the ongoing detection of these sensory responses out to 15 years old is unprecedented in the natural history of the classic GAN phenotype and provides the most striking and irrefutable signal of peripheral nervous system response to gene transfer. Responses were variable or did not show restoration in motor nerve responses or in lower extremity sensory responses. Biodistribution in one trial participant at 8.5 months post gene transfer (dose group: 3.5 × 10^13^ v.g. and was baseline AAV9 NAb seropositive) showed greater distribution along the spinal cord (range: 0.05–0.45 copies of human codon-optimized *GAN* [h*GAN*opt] per host diploid genome), but lower distribution in most regions of the brain (<0.01 h*GAN*opt per host diploid genome).^330^ This is consistent with some participants showing qualitative disease-related progression of white matter disease burden by brain magnetic resonance imaging (MRI), even post gene transfer. Histologically, there was no evidence of inflammation in the spinal cord or dorsal root ganglion in the single postmortem sample.

Overall, IT gene transfer for GAN had favorable safety at the doses evaluated and with the immune modulation approach utilized.^330^ There were no clinically significant AAV gene transfer class-related adverse events of special interest, such as thrombocytopenia, transaminitis, troponin elevation, or dorsal root ganglion toxicity.^11^^,^^76^^,^^88^^,^^331^ There is a clinical and neurophysiological sign of efficacy and target engagement following IT gene transfer for GAN despite the age and relative disease burden in trial participants at study baseline. Restoration of nerve amplitude (a marker of axonal health) occurred in a length-dependent fashion (likely concordant with extent of baseline neuropathology/neurodegeneration). Therefore, baseline nerve response, age, nerve length, and degree of neurodegeneration likely dictate the potential for and extent of efficacy following gene transfer. Future studies evaluating additional secondary and exploratory endpoints in the ongoing long-term follow-up in phase 1 participants, dosing participants at younger ages with classic GAN or at varying ages with the axonal-CMT-plus phenotype of GAN, and exploring higher doses, may further elucidate the impact of intrathecal gene transfer for GAN across the age and phenotypic spectrum.

### Canavan disease

Canavan disease (CD) is an autosomal recessive pediatric leukodystrophy, first documented by Dr. Myrtelle Canavan in 1931,^334^ characterized by severe developmental regression, seizures, lack of head control, and inability to sit, walk, and talk, ultimately leading to death at a young age.^335^^,^^336^ It took almost 6 decades to identify and clone its causative loss-of-function mutation in Aspartoacylase (ASPA), an enzyme highly expressed in oligodendrocytes in the central nervous system (CNS).^337^^,^^338^ ASPA deficiency leads to N-acetylaspartate (NAA) accumulation and vacuolization with neurodegeneration.^337^^,^^339^^,^^340^

CD soon became a model for CNS-directed gene therapy when, in 1996, the first gene therapy trial was initiated in New Zealand and later transitioned to the United States.^341^^,^^342^ At that time, recombinant AAV (rAAV) technology was still in its early stages. The initial patients received intracranial injections of non-viral liposome-based particles expressing ASPA. This phase 1 trial demonstrated safety and transient improvement in brain NAA levels, with one patient also showing signs of myelin formation on magnetic resonance imaging (MRI). One patient showed signs of functional improvement. However, the power of the trials was unable to provide information on clinically significant changes.^341^^,^^342^ Preclinical proof-of-concept data were unavailable due to the lack of a disease animal model.

In 2001, the same team conducted a phase 1/2 trial using rAAV2 for intracranial intraparenchymal delivery through six burr holes, administering a total dose of 9 × 10^11^ viral particles. This trial marked a significant milestone, as it was the first to use rAAV for CNS delivery of a therapeutic transgene.^343^ Thirteen patients, ranging from 4 to 83 months of age, received the therapy. The trial successfully demonstrated the safety of rAAV2-ASPA, though there were two serious adverse events unrelated to the viral vector (fever and brain abscess). Neurosurgical procedure-related risks, such as brain abscess, parenchymal tract hemorrhage, and subdural hematomas were noted. The trial’s secondary goals, which focused on NAA levels, brain morphology, and clinical outcomes, revealed that NAA levels did not normalize in all brain regions—likely due to the delivery method. Despite this, brain atrophy appeared to stabilize, and age at treatment seemed to influence clinical improvements such as alertness, rolling over, and rigidity. It is noteworthy that recent CNS-directed rAAV gene therapy trials have shown similar age-dependent trends. Functional outcomes, such as the Pediatric Inventory of Disability Index (PEDI), showed limited improvements, mainly in social function, but not in self-care or mobility. Additionally, no changes in receptive and expressive language were observed. Finally, seizure frequency, which can substantially impact the quality of life, appeared to be decreased.^343^

Following these early trials, CD-focused clinical gene therapy experienced a period of stagnation. During this time, advancements were made, including the engineering of mouse models, novel rAAV capsids, and improved vectors design, which enabled the next generation of rAAV-based gene therapies.^344^^,^^345^^,^^346^^,^^347^^,^^348^^,^^349^^,^^350^^,^^351^^,^^352^^,^^353^^,^^354^ These preclinical studies led to several breakthroughs: (1) the creating of three distinct CD mouse models with varying disease severity, allowing for more flexible therapeutic studies; (2) confirmation that oligodendrocyte-specific deletion of ASPA is sufficient to cause a CD-like phenotype; (3) evidence that astrocyte-restricted expression of ASPA can rescue the phenotype; (4) improved therapeutic efficacy through codon optimization; and (5) the discovery of rAAV capsids capable of crossing the blood-brain barrier, making intravenous delivery a viable, noninvasive alternative.^355^^,^^356^^,^^357^^,^^358^^,^^359^

The first human application of these next generation gene therapies occurred in 2017 as part of a single expanded access trial (NCT05317780).^360^ This expanded access was the first to assess a combined intracranial and intravenous route with a total dose of 4 × 10^14^ viral particles and concomitant immune suppression (rituximab, sirolimus, corticosteroids). The results were promising, showing long-term NAA reduction up to 86% of baseline, improved vision, and prevention of seizure onset. Myelinated fiber tract integrity showed transient improvement and signs of myelination were seen on MRI. However, the patient developed ventriculomegaly, likely due to continued brain matter loss. Cognitive function improved, albeit modestly, from a developmental level of 4 months (at 22 months of age) to 8 months (at 41 months of age). Whether the immune modulation regimen influenced the therapeutic outcome remains unclear, as the trial only involved a single participant.

Currently, two clinical trials are under way (NCT04833907, NCT04998396) (Table 3). Both trials employ self-complementary vectors with codon-optimized ASPA cDNA but differ in rAAV capsid (Olig001 vs. AAV9), promoter (shortened CBh vs. CB6), route of administration (intracranial vs. intravenous) and dose. Preclinical data in CD animal models support these approaches.^349^^,^^352^^,^^354^ Although no published data are available, interim results were presented at different meetings, recapitulating a favorable safety profile of rAAV.ASPA and immune suppression. In addition, decreased NAA levels, improved myelination, and functional recovery have been shown for both trials. Of note, the Myrtelle trial uses one dose while the ASPA Therapeutic trial uses two doses. Data presented so far are related to the Myrtelle dose and ASPA Therapeutic’s low dose. While results are not directly comparable, both current trials show substantial improvement over early efforts in 1996 and 2001.

**Table 3:** AAV clinical trials for Canavan disease

Trial	Year	NCT	Vector	Route	Dose	Sponsor
Phase 1	1996	unknown	Liposome	intracranial	unknown	New Zealand Health Research Council, Canavan Research Fund
Phase 1/2	2001	n.n.	rAAV2	intra-parenchymal	9 × 10^11^ vg total	
Single patient	2017	NCT05317780	rAAV9	intravenous & intracerebro-ventricular	4 × 10^14^ vg total	family sponsored
Phase 1/2	2021	NCT04833907	Olig001	intracerebro-ventricular	3.7 × 10^13^ total	Myrtelle
Phase 1/2	2021	NCT04998396	rAAV9	intravenous	1.32 × 10^14^ vg/kg∗, 3 × 10^14^ vg/kg∗	ASPA Therapeutics

### Aromatic l-amino acid decarboxylase deficiency

Aromatic l-amino acid decarboxylase (AADC) deficiency is an ultra-rare inherited neurological disorder arising from biallelic pathological variants in the dopa decarboxylase (*DDC*) gene.^361^^,^^362^ Deficiency of the AADC enzyme leads to an inability to synthesize dopamine and serotonin, and patients suffer from movement disorders including hypokinesia, dystonia, and oculogyric crisis that result in motor dysfunction, along with behavioral problems, autonomic dysfunction, and developmental delay. This is an ultra-rare disease and a higher prevalence is observed in the Chinese population in Taiwan,^363^ where patients die at a mean age of 3.9 years.^364^

Intraputaminal infusion of AAV2-hAADC was first utilized in the treatment of Parkinson’s disease, aiming at maintaining the therapeutic effect of levodopa.^365^^,^^366^ In 2012, Hwu et al. conducted a compassionate-use, first-in-human, clinical trial of AAV2-hAADC (now, eladocagene exuparvovec or Upstaza) in Taiwan for patients with AADC deficiency. This trial treated eight patients, for which data on four participants were published.^367^ The efficacy and safety data generated from the compassionate-use trial enabled the launch of a phase 1/2, open-label trial sponsored by the National Taiwan University Hospital. In this trial, 10 subjects were enrolled.^368^ An extension of the phase 1/2 trial (phase 2b) was then sponsored by Agilis Biotherapeutics, later acquired by PTC Therapeutics. Hwu et al. summarized the outcomes of 26 subjects who have completed 1-year evaluations.^369^ Subjects received intraputaminal infusions of AAV2-hAADC at a dose of 1.8 × 10^11^ to 2.4 × 10^11^ genome copy (GC) per person. Rapid improvements in motor and cognitive function occurred within 12 months after gene therapy and were sustained at >5-year follow-up. An increase in dopamine production was demonstrated by positron emission tomography and cerebral spinal fluid neurotransmitter analysis. Patient symptoms (mood, sweating, temperature, and oculogyric crises), growth, and caretaker quality of life improved. Although improvements were observed in all treated participants, younger age was associated with greater outcome. Brain diffusion tensor imaging (DTI) was performed in eight patients (aged 1.67–8.42 years) before and 12 months after gene therapy, showing improvement in the corticospinal tracts and the thalamic radiation and callosal fibers involving motor function.^370^ There were no treatment-associated brain injuries, and most adverse events were related to underlying disease. Post-surgery complications such as cerebrospinal fluid leakage were managed with standard of care. Most patients experienced mild to moderate dyskinesia, which resolved within a few months. Similar results were found in a study in Japan that enrolled a more diverse population, including two severely affected subjects who could wean off mechanical ventilation after treatment.^371^ Recently, an open-label trial was conducted in the United States to address the safety of the SmartFlow ventricular cannula for administering of the drug to pediatric subjects (NCT04903288).

The neuroanatomy and the function of the putamen in motor control provide rationale for targeting this brain structure. Although the dopaminergic neurons of the nigrostriatal tract reside in the substantia nigra, dopamine is secreted for neurotransmission in the putamen. Therefore, the efficacy and safety of intraputaminal infusion of low-dose AAV2-rAADC to restore dopamine synthesis in the putamen are high. This restoration leads to sustained conversion of endogenous l-DOPA into dopamine, which can be taken up by the dopaminergic nerve terminals.^372^ Reduced immunogenicity of intraparenchymal delivery of AAV2 vectors is also an advantage of the treatment.^373^ However, patients require intensive post gene-therapy rehabilitation, including an initial period of intensive rehabilitation, facilitating active movements, training for functional abilities, and cognitive and communication training.^374^

Eladocagene exuparvovec (Upstaza) was first approved in the European Union in 2022 and in the United States in 2024 (Kebilidi), and is approved in eight additional countries, including Great Britain, Brazil, Israel, and Taiwan. The availability of a treatment for such an ultra-rare disease has improved awareness of AADC deficiency, resulting in improved diagnosis, including in geographic areas such as the Middle East, where AADC deficiency was previously rarely diagnosed.^375^ Because early treatment is important, newborn screening of AADC deficiency has also been developed.^376^^,^^377^ Given Upstaza is the first approved gene therapy with direct brain delivery, the experiences derived from the use of Upstaza should facilitate the development of other gene therapies for the central nervous system in the future.

## Ocular gene therapy

The eye remains one of the most attractive targets for gene therapy for multiple reasons, including its small size and compartmentalization. This translates into restricted drug biodistribution, small dose, and ultimately a low cost of manufactured goods. It also affords the ability to assess safety and efficacy non-invasively through imaging, electrophysiologic, biophysical, and behavioral measures. The eye is also immune privileged, which reduces the risk for a host immune response. However, this “privilege” has its limits, and is more pronounced in the subretinal space than the vitreous, and even less so in the anterior chamber.^378^ It is also the first tissue for which a gene therapy was FDA approved. Voretigene Neparvovec-rzyl (Luxturna) is an AAV2-based gene therapy for *RPE65-*associated Leber congenital amaurosis (LCA2). Its approval in 2017 cemented AAV as the gold standard for gene delivery to the eye and catalyzed a plethora of additional programs. However, it is interesting to note that no additional approvals for ocular gene therapy have been achieved since. The following section summarizes clinically applied AAV-based gene therapies for both inherited retinal disease (IRD) and more prevalent blinding conditions. We focus on notable safety and efficacy outcomes that have been published.

### Leber congenital amaurosis

Leber congenital amaurosis (LCA) is a family of autosomal recessive IRDs that cause severe vision loss in children. There are more than 20 different genes associated with LCA playing a role in retinal development and physiology.^379^ *RPE65*-associated LCA2 was the first disease for which a licensed gene therapy in the United States was developed. RPE65 metabolizes all-*trans*-retinyl ester to 11-*cis*-retinol in the retinal pigment epithelium (RPE), which is needed by photoreceptors (PRs) to replenish visual pigment (11-*cis* retinal). Its absence impairs the ability of PRs to respond to light, leading to their progressive degeneration. While three initial first-in-human studies of AAV2-RPE65 were conducted, two of these initial trials demonstrated only modest and nonsustained vision improvements.^380^^,^^381^ In contrast, voretigene neparvovec (Luxturna, Spark Therapeutics) demonstrated significant improvements in retinal sensitivity and functional vision with a clean safety profile that was sustained long-term.^382^ However, while efficacy was sustained, long-term follow-up revealed that a percentage of patients treated with voretigene neparvovec developed chorioretinal atrophy within and around the SRI bleb,^383^ a finding perhaps attributed to overexpression of RPE65 in the RPE. Nonetheles, long-term efficacy observations with voretigene neparvovec relative to the other investigational AAV2-RPE65 vectors highlights the potential for divergent clinical outcomes, despite approaches. Last, voretigene neparvovec, was the first FDA-approved AAV vector, and established both a regulatory and commercialization pathway for AAV vectors.

*GUCY2D*-associated LCA1 affects more than double the number of patients who have LCA2. *GUCY2D* encodes retinal guanylate cyclase (retGC1), a protein expressed in PRs that plays a role in the recovery phase of phototransduction. After demonstrating proof of concept in multiple animal models,^384^^,^^385^^,^^386^^,^^387^ Sanofi and Astena Therapeutics conducted a phase 1/2 clinical trial (NCT03920007) evaluating a subretinally delivered AAV5 vector containing the photoreceptor-specific hGRK1 promoter and *GUCY2D* coding sequence.^388^^,^^389^^,^^390^ Significant improvements in both retinal sensitivity and visually guided behavior were observed in treated patients, and no signs of chorioretinal atrophy have emerged.

LCA5 is caused by mutations in lebercillin (*LCA5*), which encodes a protein expressed in the photoreceptor connecting cilium. Opus Genetics is in early testing stages for gene supplementation by subretinally delivered AAV8-LCA5 in a phase 1/2 trial (NCT05616793).

LCA10 is a prevalent form of LCA caused by mutations in *CEP290*, which also encodes a protein expressed in the PR connecting cilium. It is a relatively large gene whose coding sequence does not fit inside a standard AAV vector. Multiple alternative approaches have been pursued to treat this specific disease. First, Editas Medicine targeted the common deep intronic cryptic splice site, IVS26, mutation with “EDIT-101,” a subretinally delivered AAV5 vector expressing Cas9 via the PR-specific hGRK1 promoter, and guide RNAs targeting editing to regions flanking IVS26 (NCT03872479). A fraction of patients (four of 14) showed improvements in best corrected visual acuity (BCVA) ≥15 letters.^391^ Ocugen is currently testing a gene agnostic approach in LCA10 patients. “OCU400” is an AAV5 vector containing coding sequence for NR2E3, a nuclear hormone receptor and transcription factor that has shown therapeutic benefits (prevention of degeneration and maintenance of function) in multiple mouse models of retinitis pigmentosa. In their ongoing phase 1/2 trial, subretinally delivered OCU400 is being tested in patients with CEP290-LCA1, as well as multiple forms of autosomal dominant retinitis pigmentosa (NCT05203939).

### Choroideremia

X-linked choroideremia (CHM) is associated with mutations in the CHM gene, which encodes the Rab escort protein-1 (REP-1), involved in intracellular trafficking of various lipid membrane-bound structures. Patients present in the first decade with nyctalopia, and progress to peripheral vision loss in their teens. Although they maintain central vision until the fifth to seventh decade, they eventually experience rapid loss of central vision and loss of RPE, PRs, and choroid. Multiple clinical trials employing subretinally delivered AAV2-CBA-REP-1 have taken place (NCT01461213, NCT02077361, NCT02553135, NCT02671539, NCT03507686, and NCT03496012). However, neither Biogen nor Spark achieved levels of improvement in BCVA for approval.^392^^,^^393^^,^^394^^,^^395^^,^^396^ 4DMT is evaluating an intravitreally delivered R100-REP-1 in CHM patients (NCT04483440).

### X-linked retinoschisis

Mutations in *RS1* are associated with X-linked retinoschisis (XLRS), an indication that presents in males in early childhood and is characterized by splitting of the retina (schisis) and loss of retinal function. Despite the early presentation, XLRS patients do not lose PR structure until their fifth to sixth decade, allowing a wide treatment window for intervention. AGTC and the NEI-sponsored phase 1/2 trials utilize intravitreally injected AAV2(tYF)-CBA-RS1 (NCT02416622) or AAV8-RS1p-RS1 (NCT02317887), respectively. Neither showed signs of biological activity, and patients in both trials exhibited inflammation. Atsena Therapeutics is now testing a subretinally delivered AAV.SPR-hGRK1-RS1 in a phase 1/2 trial (NCT05878860). They are investigating whether their capsid may permit safe, peripheral placement of the subretinal bleb, away from the macular schisis, as well as effective gene delivery to the central retina.^397^

### Leber hereditary optic neuropathy

Leber hereditary optic neuropathy (LHON) is caused by mutations in the mitochondrial genes that encode the NADH dehydrogenase subunits involved in oxidative phosphorylation (*ND1*, *ND4*, *ND6*). Patients present with preferential loss of retinal ganglion cells (RGCs) that may lead to sudden loss of central vision. Multiple trials have focused on gene supplementation of *ND4*, the most commonly associated gene (NCT02161380, NCT02064569, NCT02652767, NCT02652780, NCT03293524, NCT01267422, NCT02161380). All but one have utilized intravitreally injected AAV2-CMV-ND4,^398^^,^^399^^,^^400^ and another one used intravitreally delivered AAV2(tYF)-smCBA-ND4.^401^ Gensight Biologics’ trials showed sustained improvements in treated eyes, but results were complicated by BCVA improvements seen in the untreated, contralateral eyes. Positive results were seen after bilateral treatment,^399^ and the gene therapy is currently under review by the Agence Nationale de Sécurité du Médicament et des Produits de Santé (ANSM) for the early access (AAC) program in France.

### Achromatopsia

Achromatopsia (ACHM) is associated with mutations in several genes, including *CNGA3* and *CNGB3*, which encode subunits of the cyclic nucleotide gated ion channels in cone photoreceptors. In their absence, cones cannot adequately respond to cGMP levels, and their phototransduction capabilities are severely impaired. Patients present in early childhood with loss of color vision, photophobia, and reduced BCVA. AGTC-sponsored phase 1/2 trials for *CNGA3-* (NCT02935517) and *CNGB3-* (NCT02599922) ACHM utilizing subretinally injected AAV2tYF containing the cone-specific PR1.7 L-opsin promoter driving either *CNGA3* (AGTC-402) or *CNGB3* (AGTC-401). Safety was established at low and mid doses, but dose-limiting toxicity was observed at high doses in children.^402^ Neither led to clinically meaningful improvements in BCVA or reduction in photophobia.^403^ An academic phase 1/2 trial evaluated subretinally delivered AAV8 containing the cone-specific arrestin-3 (ARR3) promoter driving CNGA3 coding sequence (NCT02610582). The treatment was generally well tolerated and some improvements in cone function were observed via secondary endpoints.^404^ Meira GTx similarly tested a subretinally injected AAV8 vector containing a cone-specific green opsin promoter (hG1.7) and *CNGA3* (NCT03758404), or a cone arrestin promoter and *CNGB3* (NCT03001310). Only results of the CNGB3 trial have been presented and they revealed no consistent efficacy signal and dose-responsive inflammation.^405^

### Stargardt disease

Mutations in *ABCA4* are associated with Stargardt disease (STGD), an early-onset form of macular degeneration. Due to the large size of the gene’s coding sequence, alternative strategies beyond standard gene replacement have been pursued. Nanoscope is currently evaluating an optogenetics-based approach in a phase 2 trial (NCT05417126) employing intravitreally injected AAV2 containing the bipolar specific mGluR6 promoter and a “multi-characteristic opsin” (MCO-010).^406^ Their goal is to re-animate bipolar cells in patients who have already lost their native light-sensitive photoreceptor cells. Additionally, Ocugen is sponsoring a phase 1/2 trial (NCT05956626) evaluating a subretinally delivered AAV5 vector containing RORA (Retinoic Acid Related Orphan Receptor A that is based on the nuclear hormone receptor regulating pathways linked to STGD pathology). Ascidian Therapeutics is sponsoring a phase 1/2 trial (NCT06467344) investigating a subretinally delivered AAV8 vector containing an RNA exon editor designed to correct STGD-causing mutations and stop or slow disease progression. Last, ViGeneron is using a dual AAV vector technique based on mRNA *trans*-splicing to enable reconstitution of the full-length ABCA4 transcript.^407^ They recently received IND clearance to evaluate their dual vector approach in a phase 1/2 trial.

### Retinitis pigmentosa

Retinitis pigmentosa (RP) is a clinically and genetically heterogeneous disease that causes loss of vision beginning with the peripheral retina that progresses to the central retina, leading to tunnel vision and eventually complete blindness. Onset of symptoms can be in childhood and are often associated with difficulty seeing at night, but clinical presentation is often later than LCA. Approximately 100 genes have been associated with RP, and only a fraction of these have been interrogated with gene therapy.

*MERTK*-associated RP is a recessively inherited form of the disease that results from a failure of the RPE to properly phagocytose PR outer segments, resulting in their buildup and ultimately, retinal degeneration. Subretinally delivered AAV2 containing the RPE-specific VMD2 promoter and MERTK coding sequence was tested in phase 1/2 clinical trials (NCT01482195). Improvements in best corrected visual acuity (BCVA) were noted in some patients, but the effect was transient.

*RPGR* is the gene most commonly associated with X-linked RP. It encodes a protein expressed in the PR connecting cilium.^408^^,^^409^ Nighstar/Biogen previously evaluated a subretinally delivered AAV8 vector containing codon-optimized RPGR. Their phase 2/3 trials (NCT03584165) did not meet the FDA-required efficacy threshold (improvement of at least 7dB sensitivity at five prespecified retinal loci as determined by microperimetry). Beacon Therapeutics (formerly AGTC Inc.) is now testing subretinally delivered AAV2(tYF)-hGRK1-RPGR (AGTC-501) in a phase 2 trial (NCT04850118). Meira GTx/Janssen is testing subretinally delivered AAV5-RPGR in a phase 3 trial (NCT04671433).

*RLBP1*-associated RP is recessively inherited and is caused by defects in the visual cycle (the conversion of 11-*trans*-retinal to 11-*cis* retinal). Novartis sponsored a phase 1/2 trial testing a subretinally delivered AAV8-RLBP1 (NCT03374657) that showed improvements in dark adaptation kinetics and resolution of disease-related deposits (in the three patients who had them at baseline). However, no improvements in BCVA, low light visual acuity (LLVA), microperimetry, or visual field were observed. Two recessive forms of RP are caused by mutations in two proteins, PDE6A and PDE6B, that together form cyclic-GMP (cGMP)-specific phosphodiesterase. Mutations in both genes impair cGMP breakdown in rods, leading to their degeneration. Investigators at Munich and Tubingen evaluated subretinally delivered AAV8-PDE6A. Despite promising preclinical data in mouse and dog models, no improvement in visual function or preservation of retina was observed in patients following treatment (NCT04611503). Coave Therapeutics is actively sponsoring a phase 1/2 trial investigating subretinally delivered AAV5-PDE6B (NCT03328130).

Gene agnostic approaches are also being evaluated in patients with advanced RP. As mentioned above, Ocugen is evaluating subretinally delivered AAV5-CBA-NR2E3 in autosomal dominant RP (adRP) patients (NCT05203939). Optogenetic approaches are also being tested for their ability to “re-animate” downstream retinal neurons after photoreceptors have degenerated in late-stage RP patients. Examples include Abbvie’s phase 1/2 trial evaluating intravitreally delivered AAV2-Channelrhodopsin-2 (RST-101) to re-animate RGCs (NCT02556736), Nanoscope Therapeutics’ phase 2b trial evaluating an intravitreally delivered AAV2 vector containing mGluR6 and a “multi-characteristic opsin” (MCO-010) (NCT04945772), and Bionic Sight’s phase 1/2 trial testing intravitreal AAV2-Chronos paired with a device (worn like glasses). Both Nanoscope and Bionic Sight have issued press releases^410^ indicating a percentage of treated patients had increased BCVA and/or regained the ability to discriminate objects.

### Age-related macular degeneration, geographic atrophy, and diabetic macular edema

AAV has also been evaluated for its ability to confer therapy in patients with much more prevalent blinding conditions such as age-related macular degeneration (AMD), geographic atrophy (GA), and diabetic macular edema (DME). AMD is the leading cause of blindness in the elderly and is characterized by impairment of the central visual field. Patients with “dry” AMD present with cellular debris (drusen) in their macula that gradually kills PRs/impairs vision. Patients with advanced dry AMD progress to geographic atrophy (GA), which is characterized by irreversible loss of PRs, RPE, and choriocapillaris. Mutations in genes associated with the complement system are strongly associated with AMD risk. As such, several gene therapies have focused on this pathway. Janssen Research and Development has sponsored trials utilizing intravitreally injected AAV2 containing chimeric CMV-chicken beta-actin promoter (CAG or CBA) driving CD59 in both wAMD (NCT03585556) and dryAMD/GA (NCT03144999) patients. CD59 is an inhibitor of the complement membrane attack complex (MAC).^411^ Gyroscope/Novartis evaluated a subretinally injected AAV2 vector containing CAG driving complement factor inhibitor (CFI) (NCT03846193), but the trial was discontinued due to weak efficacy data.^412^ Patients with “wet” AMD present with growth of new blood vessels under the macula that often leak and can eventually occlude vision. As such, gene therapies have focused on inhibiting neovascularization with the hope of finding a one-time treatment that precludes patients from needing monthly injections of vascular endothelial growth factor (VEGF) inhibitors. The Lions Eye Institute sponsored a phase 1 trial (NCT01494805) using subretinal AAV2 containing soluble fms-like tyrosine kinase-1 (-sFLT-1), a molecule that binds to VEGF and placental growth factor (PlGF) and prevents angiogenesis. Treated patients displayed either maintained or improved vision and needed fewer ranibizumab treatments.^413^ Genzyme’s phase 1 trial (NCT01024998) evaluated intravitreally injected AAV2-CBA-sFLT-1 and demonstrated safety, but efficacy was hampered in some patients due to preexisting neutralizing antibodies (NAbs).^414^ Adverum used intravitreal AAV7m8-CMV-Aflibercept in a phase 1 trial (NCT03748784). No serious adverse events have been reported, BCVA in patients has been maintained, and over 80% of patients have not needed an anti-VEGF injection up to 92 weeks after their treatment.^415^^,^^416^ This trial also revealed a correlation between preexisting NAbs and the need for continued anti-VEGF treatments and has progressed to phase 2 (NCT05536973). The same vector was used by Adverum in a phase 2 trial (NCT04418427) to treat diabetic macular edema (DME), a condition that arises from leakage of damaged blood vessels in patients with diabetic retinopathy. However, the study was discontinued due to safety concerns. 4DMT is evaluating intravitreally injected AAV with their R100 capsid containing both aflibercept and a VEGF-C inhibitory RNAi to inhibit VEGF A, B, C and placental growth factor (PlGF), in a phase 1/2 trial for wet AMD (NCT05197270). Finally, after a phase 1/2 trial sponsored by Regenxbio (NCT03066258), Regenxbio/Abbvie began phase 2b/3 (NCT03066258) and phase 3 (NCT05407636) trials evaluating subretinally delivered AAV8-CBA-antiVEGFfab in wet AMD patients. Phase 2b/3 results showed a relatively clean safety profile and durable treatment effect. The success of any gene therapy in the wAMD space ultimately will hinge on whether it provides an improvement over the standard of care.

## Summary

In this review of clinical AAV gene therapy efforts, our goal has been to capture the most comprehensive and up-to-date information in the rapidly advancing field of AAV gene therapy. Each of the contributors are leading experts in their area of focus for the review, yet there are no doubt gaps in our knowledge or some areas where significant advances are not yet public knowledge or clinical data are imminent. We hope that the extensive detail provided in this review captures the breath of topics where gene therapy is changing medical practice for a range of genetically defined disorders and giving rise to the field of genetic medicine.

What started as a very niche area of virology for investigators at NIH and a few academic intuitions, the field of AAV biology and gene therapy has resulted in over 6,000 publications from 25,000 authors since 1991.^417^ Many of these initial seminal observations were made by University of Florida faculty, where the concept of using AAV as a therapeutic was developed. The rapid growth and worldwide dissemination of the technology for both *in vivo* human gene therapy and as a fundamental tool in molecular genetics is a testament to the utility of the platform that has evolved over the last 3 decades.

Despite this long history, the next wave of innovation is just now emerging with great anticipation for novel bioengineered AAV vector capsids through directed evolution as well as payloads that can augment or replace gene function, silence or edit genes, as well as modify epigenetic marks and even edit single bases or modify repeat expansions. As the field matures, we look forward to the next generation of scientists and clinicians to harness these tools to advance *in vivo* gene editing, make further inroads developing gene therapy for ultra-orphan conditions as well as large population disorders, and solve barriers to access worldwide.

## Acknowledgments

The authors wish to acknowledge the enormous contributions from the broader scientific community, which generated the primary data in this review. We would also like to acknowledge the public trust required for study participants to enter into an investigational study and further our collective knowledge of the risks, benefits, and therapeutic potential of *in vivo* gene therapy.

## Author contributions

Each author was responsible for writing, review, and correction of the manuscript based on current literature and personal knowledge.

## Declaration of interests

B.J.B. has received research support from 10.13039/100014943Sarepta Therapeutics and 10.13039/100004319Pfizer and is a member of the Global Pompe Advisory Board supported by 10.13039/100004339Sanofi. B.J.B. has received consulting fees from Amicus Therapeutics, Rocket Pharma, and Tenaya. B.J.B. is a co-founder of Ventura Life Sciences, LLC. The University of Florida is entitled to licensing revenue related to AAV technology. B.J.B. is an uncompensated member of the MDA Board of Directors. B.J.B. has grant funding from R01-HD052682 and U01-NS116752-01A1. R.S.F. declares personal compensation for advisory board participation from Astellas, Biogen, Dyne, Genentech, Ionis, Italfarmaco, Novartis, ReveraGen, Roche, Sarepta, and Scholar Rock; data safety monitoring board participation for a Sarepta-sponsored study on Duchenne muscular dystophry and 10.13039/100007520Nationwide Children's Hospital study on SMA; research funding from 10.13039/100005614Biogen, Dyne, 10.13039/100004328Genentech, Genethon, 10.13039/501100014383Italfarmaco, 10.13039/100004337Roche, 10.13039/100014943Sarepta, and Scholar Rock; editorial fees from Elsevier for co-editing a neurology textbook; and license fees from the 10.13039/100006458Children's Hospital of Philadelphia. R.S.F. is affiliated with an institution (St. Jude Children’s Research Hospital) that receives funds for an SMA disease registry (ISMAR). R.S.F. has received funding to support clinical trial participation to his institution from 10.13039/100005614Biogen, Dyne, 10.13039/100004328Genentech, Genethon, 10.13039/501100014383Italfarmaco, 10.13039/100004337Roche, 10.13039/100014943Sarepta, and Scholar Rock. M.A.W. receives clinical trial support from 10.13039/100004336Novartis and 10.13039/100014943Sarepta Therapeutics and serves as a consultant for Sarepta Therapeutics. AHRQ-PCORI funded the PEDSnet Scholars Training Program 5K12HS026393-03, which is a national faculty development program that trains individuals in the competencies of learning health systems science. N.E.J. has received grant funding from 10.13039/100000065NINDS (R01NS104010, U01NS124974), 10.13039/100006108NCATS (R21TR003184), 10.13039/100000030CDC (U01DD001242), and the 10.13039/100000038FDA (7R01FD006071). N.E.J. receives royalties from the CCMDHI and the CMTHI. N.E.J. receives research funds from 10.13039/100004336Novartis, 10.13039/100007723Takeda, PepGen, 10.13039/100013995Sanofi Genzyme, Dyne, 10.13039/100011022Vertex Pharmaceuticals, Fulcrum Therapeutics, AskBio, ML Bio, and 10.13039/100014943Sarepta. N.E.J. has provided consultation for Arthex, Angle Therapeutics, Juvena, Rgenta, PepGen, AMO Pharma, Takeda, Design, Dyne, AskBio, Avidity, and Vertex Pharmaceuticals. N.E.J. has stock options in Juvena, Angle Therapeutics, and Myogene Therapies. J.W.R. is a consultant for AskBio, Bayer, Bristol Myers Squibb, and Merck. B.G. is the principal site investigator for clinical trials of gene therapy that are funded by Rocket Pharma. B.G. chairs two DSMB committees overseeing clinical trials of gene therapy sponsored by Tenaya. B.G. has served as a consultant for Astellas, AstraZeneca, Bristol Myers Squibb, and Tenaya. Rocket Pharma provides support to UCSD for clinical trials of gene therapy. B.G. serves as a chair of the DSMB for two clinical trials in gene therapy funded by Tenaya. B.G. has also received honoraria for consulting activities with Astellas, AstraZeneca, Bristol Myers Squibb, and Tenaya. L.L. is an inventor of the patent application EP25382006.2 entitled “Method for improving cardiac function in arrhythmogenic right ventricular cardiomyopathy type 5.” E.L.-P. is an inventor of the patent application EP25382006.2 entitled “Method for improving cardiac function in arrhythmogenic right ventricular cardiomyopathy type 5.” E.L.-P. has received support from the 10.13039/100018703European Innovation Council (Award Number: 101115416 - Enabling advances in diagnosis, patient stratification and treatment for dilated cardiomyopathy patients and families [DCM-NEXT]). M.C. has received research support from the 10.13039/100002108Friedreich's Ataxia Research Alliance. M.C. and B.J.B. are co-founders of Ventura Life Sciences, LLC. The University of Florida is entitled to licensing revenue related to Pompe disease inventions. M.C. has grant funding from R01-HD052682 and U01-NS116752-01A1. R.A.-N. is an advisor to LatusBio and serves on a DSMB for AskBio. Y.-H.C. declares receiving compensation for consultations and serving as an advisory board member for PTC Therapeutics, Roche, and Novartis. S.L.B. is a co-founder and consultant for Atsena Theraepeutics and has patents related to technologies described in this manuscript. S.E.B. is a co-founder and consultant for Atsena Theraepeutics and has patents related to technologies described in this manuscript. S.E.B. has funding from the 10.13039/100000053National Eye Institute (R01 EY024280), the 10.13039/100001116Foundation Fighting Blindness, 10.13039/100002108Friedreich's Ataxia Research Alliance, Stiftung für Medizininnovationen, and Atsena Therapeutics. L.A.G. is on the Scientific Advisory Board of Form Bio, is a consultant for CSL Behring, Myogene, Pfizer, Regeneron, and Spark Therapeutic and holds patents related to data discussed in this work.
